# Mass, phylogeny, and temperature are sufficient to explain differences in metabolic scaling across mammalian orders?

**DOI:** 10.1002/ece3.2555

**Published:** 2016-10-24

**Authors:** Eva Maria Griebeler, Jan Werner

**Affiliations:** ^1^ Department of Evolutionary EcologyInstitute of ZoologyJohannes Gutenberg UniversityMainzGermany

**Keywords:** Allometry, body mass, body temperature, constraints, macrophysiology, phylogeny

## Abstract

Whether basal metabolic rate‐body mass scaling relationships have a single exponent is highly discussed, and also the correct statistical model to establish relationships. Here, we aimed (1) to identify statistically best scaling models for 17 mammalian orders, Marsupialia, Eutheria and all mammals, and (2) thereby to prove whether correcting for differences in species’ body temperature and their shared evolutionary history improves models and their biological interpretability. We used the large dataset from Sieg et al. (*The American Naturalist* **174**, 2009, 720) providing species’ body mass (BM), basal metabolic rate (BMR) and body temperature (*T*). We applied different statistical approaches to identify the best scaling model for each taxon: ordinary least squares regression analysis (OLS) and phylogenetically informed analysis (PGLS), both without and with controlling for *T*. Under each approach, we tested linear equations (log‐log‐transformed data) estimating scaling exponents and normalization constants, and such with a variable normalization constant and a fixed exponent of either ⅔ or ¾, and also a curvature. Only under temperature correction, an additional variable coefficient modeled the influence of *T* on BMR. Except for Pholidata and Carnivora, in all taxa studied linear models were clearly supported over a curvature by AICc. They indicated no single exponent at the level of orders or at higher taxonomic levels. The majority of all best models corrected for phylogeny, whereas only half of them included *T*. When correcting for *T*, the mathematically expected correlation between the exponent (*b*) and the normalization constant (*a*) in the standard scaling model *y* = *a x* ^*b*^ was removed, but the normalization constant and temperature coefficient still correlated strongly. In six taxa, *T* and BM correlated positively or negatively. All this hampers a disentangling of the effect of BM,* T* and other factors on BMR, and an interpretation of linear BMR‐BM scaling relationships in the mammalian taxa studied.

## 1 Introduction

For the last two centuries, the relationship between body mass and metabolic rate has been of great interest. The relationship between metabolic rate (MR) and body mass (BM) is typically expressed as a power function (*MR* = *a BM* ^*b*^) with an exponent *b* and a normalization constant *a*. A linear scaling model results from a log‐log transformation of the power function (log_10_(*MR*) = log_10_(*a*) + *b* log_10_(*BM*)). Here, *b* is the slope and log_10_(*a*) the intercept of a straight line.

While some studies support that metabolic rate scales in proportion to *BM* ^⅔^ (Heusner, 1991; Rubner, 1883; White & Seymour, 2003), others reject the ⅔ exponent. The latter studies suggest an exponent of ¾ (Brown, Gillooly, Allen, Savage, & West, 2004; Kleiber, 1932; Savage et al., 2004) or that the exponent varies between taxa and depends on physiology, environment and taxonomy (Glazier, 2005; McNab, 2008, 2009; Sieg et al., 2009; White, 2010; White, Phillips, & Seymour, 2006).

Within the field of ecology, interest in metabolic scaling has increased greatly during the last decade due to the Metabolic Theory of Ecology (MTE; Brown et al., 2004). The MTE relies on a general ¾ power scaling of resting (basal) metabolic rate with body mass. It utilizes an Arrhenius approach to model differences in metabolic rates of similar‐sized species that result from temperature effects on underlying biochemical reactions. The MTE provides a mechanistic theory for a quarter‐power scaling—the West, Brown and Enquist resource distribution network model (WBE; West, Brown, & Enquist, 1997)—and links the metabolic rate of organisms to their biology and the ecology of populations, communities and even ecosystems.

In the context of the MTE, Kolokotrones, Savage, Deeds, and Fontana (2010) reported a convex curvilinear metabolic scaling for mammals (log‐log plot), and thus an increasing scaling exponent with increasing body mass. This pattern was corroborated by other authors (Capellini, Venditti, & Barton, 2010; Clarke, Rothery, & Isaac, 2010; Isaac & Carbone, 2010; Müller et al., 2012). Kolokotrones et al. (2010) argued that the curvature resolves the controversy surrounding the scaling exponent (¾ vs. ⅔ power) for mammals, that the curvature demands a modification of the WBE, and that the curvature explains the upper limit of animal body mass in mammals (the blue whale).

Several authors have demonstrated differences in basal metabolic rates of mammals at the level of single orders (Capellini et al., 2010; Clarke et al., 2010; Hayssen & Lacy, 1985; Isaac & Carbone, 2010; McNab, 2008; White & Seymour, 2004) and at higher taxonomic levels (Duncan, Forsyth, & Hone, 2007; Müller et al., 2012; Sieg et al., 2009). Orders dominated by larger species have larger scaling exponents than orders dominated by smaller species (Clarke et al., 2010; Duncan et al., 2007; Glazier, 2005). The majority of studies on metabolic scaling in mammalian taxa mainly focused on the variability seen in scaling exponents, whereas the variability in normalization constants was often ignored (but see Duncan et al., 2007; Sieg et al., 2009; Isaac & Carbone, 2010). Statistical approaches used to assess differences in scaling exponents of mammalian orders considerably differ between studies, making a quantitative comparison of scaling relationships found problematic. For example, authors used ordinary least squares regression analysis (e.g., Hayssen & Lacy, 1985; White & Seymour, 2004), applied regression analysis without fully correcting for a shared evolutionary history of species (e.g., McNab, 2008), used phylogenetically informed regression analysis (e.g., Clarke et al., 2010; Sieg et al., 2009; White, 2011; White, Blackburn, & Seymour, 2009), applied ANCOVA (e.g., McNab, 2008), or used linear mixed‐effect models to assess differences between orders (e.g., Isaac & Carbone, 2010). To the best of our knowledge, only one study exists that simultaneously corrects for phylogeny and body temperature at the order level and studies a broader taxonomic range in mammals (Clarke et al., 2010). The most important reason given by authors for ignoring the shared evolutionary history of species is that physiological “performance” characteristics of species such as metabolism have been repeatedly described as sensitive to environmental conditions (e.g., food availability and quality, climate, altitude, an island or continental distribution, the use of torpor and rate of reproduction; for a summary, see McNab, 2012) and thus do not reflect ancestral relationships. The pattern that and how basal metabolic rate, body temperature and body mass correlate in all mammals and in mammalian taxa is complex, which questions whether correcting by body temperature is indeed useful in scaling analyses. For Carnivora, Erinaceomorpha and Artiodactyla (Clarke & Rothery, 2007), a decrease in body temperature with increasing body mass has been shown, and an increase in all mammals (Griebeler, 2013), Eutheria (Griebeler, 2013), Marsupialia (Clarke & Rothery 2007; Griebeler, 2013) and Chiroptera (Clarke & Rothery 2007). For Insectivora, basal metabolic rate and body temperature correlate positively, while at a lower taxonomic level (subfamily, family) this correlation considerably diminishes (McNab, 2006).

In this study, we established metabolic scaling relationships for 17 mammalian orders, for Marsupialia, Eutheria and all mammals. Our aims were (1) to identify best scaling models for taxa, and (2) thereby to prove whether a correction for differences in species’ body temperature and the shared evolutionary history indeed statistically improves these best scaling models and their biological interpretability. We therefore used the large dataset from Sieg et al. (2009) comprising 695 mammalian species. It provides information on species’ body mass, basal metabolic rate, and body temperature. For 519 of these, phylogenetic information was also available to us. The total dataset of Sieg et al. (2009) was analyzed by Kolokotrones et al. (2010), and these authors additionally studied a similar dataset from McNab (2008). The dataset of McNab (2008), however, has fewer species and fewer body temperatures than the dataset of Sieg et al. (2009). Nevertheless, both datasets show a large overlap in species.

We performed linear and quadratic (curvature; Capellini et al., 2010; Clarke et al., 2010; Isaac & Carbone, 2010; Kolokotrones et al., 2010; Müller et al., 2012) least squares regression analyses on log‐log‐transformed data to identify the best scaling relationship for mammalian orders, Marsupialia, Eutheria, and all mammals. In particular, we tested three linear models (slope and intercept are estimated, fixed slope of 0.75 or of 0.67 with an estimated intercept) and one quadratic model for these taxa under four statistical scenarios, yielding a total of 16 models considered for each taxon. The four statistical scenarios were (1) ordinary least squares regression analysis (OLS) without and (2) with temperature correction, and (3) phylogenetic generalized least squares regression analysis (PGLS; Pagel, 1997, 1999; Freckleton, Harvey, & Pagel, 2002) without and (4) with temperature correction. For each of the studied taxa, we assessed the overall best model out of the 16 models considered from their AICc values. This enabled us to assess whether a correction for phylogeny and body temperature improves the scaling relationship obtained for a taxon, and in the case that a linear model worked best whether its slope supports a ¾ or ⅔ power scaling or none of both. Finally, for each statistical scenario, we explored across orders correlations between their regression coefficients of linear scaling models estimating the exponent, normalization constant, and if applicable of the temperature term. For standard allometric relationships (*y *= *a x* ^*b*^), a correlation between the exponent and normalization constant is mathematically expected (Gould, 1966; White & Gould, 1965). Thus, we extended the empirical study of Sieg et al. (2009) who reported correlations between exponents and normalization constants when examining heterogeneous taxonomic levels of mammals. With this analysis, we aimed to figure out the effect of temperature correction on this built‐in correlation between exponents and normalization constants in order to disentangle the effect of body mass, temperature, phylogeny and other factors on scaling relationships of studied mammalian taxa.

## 2 Material and Method

### 2.1 Dataset analyzed

**Figure 1:**
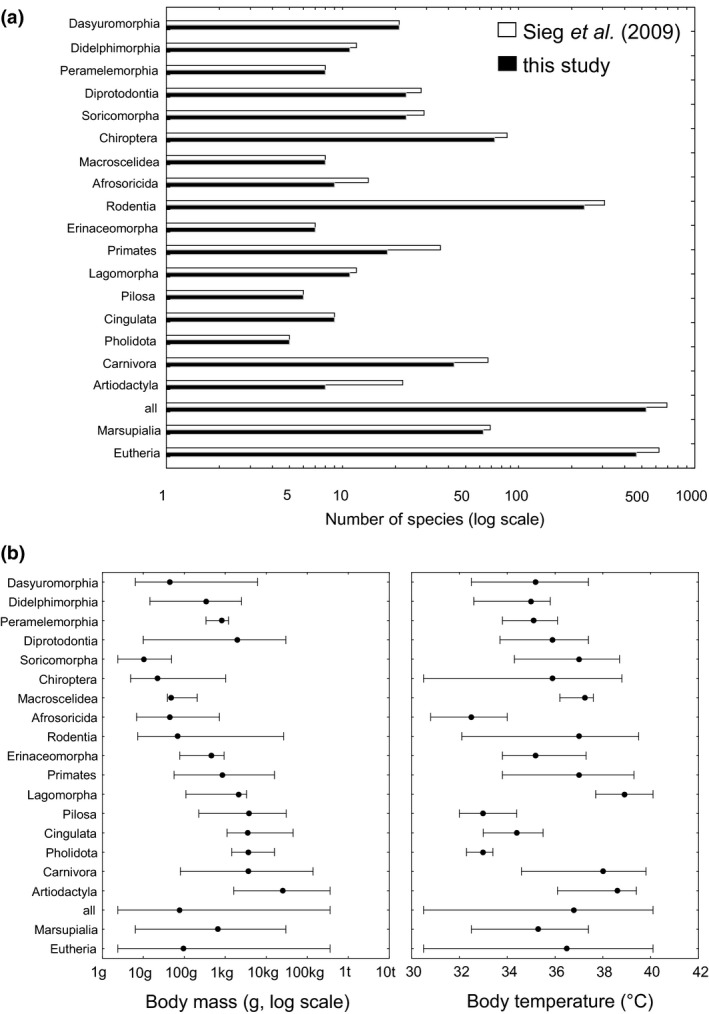
(a) Sample sizes of mammalian taxa studied here and covered in the original dataset of Sieg et al. (2009). (b) Body mass and temperature range of mammalian taxa studied by us, medians (circle) and ranges (whiskers) are shown

For our large‐scale analyses on scaling in mammalian basal metabolic rate (BMR), we used the dataset on body mass (BM), BMR and body temperatures (*T*) published by Sieg et al. (2009). This dataset covers a total of 695 species from 27 orders. We used a subset of 519 species from this dataset for which information on BM, BMR and *T* was given therein and for which phylogenetic information was also available. The 519 species comprise 17 orders which are represented by at least five species (Figure 1). These were four marsupialian orders (Dasyuromorphia, Didelphimorphia, Diprotodontia, Peramelemorphia), and 13 eutherian orders (Afrosoricida, Artiodactyla, Carnivora, Chiroptera, Cingulata, Erinaceomorpha, Lagomorpha, Macroscelidea, Pholidota, Pilosa, Primates, Rodentia, Soricomorpha). Due to the lack of information on species’ body temperatures or phylogeny, we had to exclude ten orders in our analysis covered in the original dataset of Sieg et al. (2009) (Cetacea, Hyracoidea, Microbiotheria, Monotremata, Notoryctemorphia, Perissodactyla, Proboscidea, Scandentia, Sirenia, Tubulidentata).

### 2.2 Phylogeny of mammals used

For phylogenetic correction, we used the fullest available mammalian phylogeny published by Bininda‐Edmonds et al. (2007). Following Sieg et al. (2009), we eliminated misspellings and taxonomical inconsistencies in this super tree. The latter correction was needed, because the tree is based on the second edition of the encyclopedia “Mammal Species of the World,” and the dataset of Sieg et al. (2009) refers to the third edition (Wilson & Reader, 2005).

### 2.3 Statistical analyses

Four equations linking BMR (log_10_‐transformed) to BM (log_10_‐transformed) in mammals, marsupials, eutherians, and in each of the 17 mammalian orders are basal to our study. They implement controversial current hypotheses on metabolic scaling in mammalian taxa and refer to empirical approximations (Hulbert, 2014). In all equations, ε is the error term.

The first equation models a standard linear scaling relationship in which the slope (β_1_) and intercept (β_0_) are estimated.

(1) $${\text{log}}_{10}\left(\text{BMR}\right)=\beta_{0}+\beta_{1}{\text{log}}_{10}\left(\text{BM}\right)+\varepsilon$$

Equation (1) is consistent with observations that the scaling exponent varies between taxa due to differences in species’ physiology, environment, and taxonomy (Glazier, 2005; McNab, 2008, 2009; Sieg et al., 2009; White, 2010; White et al., 2006). Two further equations that we used are linear scaling models that either have a fixed slope of 0.75 or a fixed slope of 0.67. Here, only the intercepts are estimated.

(2) $${\text{log}}_{10}\left(\text{BMR}\right)=\beta_{0}+0.75{\text{log}}_{10}\left(\text{BM}\right)+\varepsilon$$

(3) $${\text{log}}_{10}\left(\text{BMR}\right)=\beta_{0}+0.67{\text{log}}_{10}\left(\text{BM}\right)+\varepsilon$$

Equation (2) models a quarter‐power scaling (Kleiber, 1932; Brown et al., 2004; WBE), and equation (3) a geometric scaling of basal metabolic rate (Rubner, 1883, surface to volume ratio).

The last equation is a quadratic scaling model.

(4) $$\begin{array}{cc} {\text{log}}_{10}\left(\text{BMR}\right) & =\beta_{0}+\beta_{1}{\text{log}}_{10}\left(\text{BM}\right)+\beta_{2}{\left({\text{log}}_{10}\left(\text{BM}\right)\right)}^{2}+\varepsilon \\ & =\beta_{0}+\left\{\beta_{1}+\beta_{2}{\text{log}}_{10}\left(\text{BM}\right)\right\}{\text{log}}_{10}\left(\text{BM}\right)+\varepsilon \end{array}$$

It models that the scaling exponent is a nonconstant value that increases or decreases with BM (convex curvature with β_2_ > 0 suggested by Kolokotrones et al., 2010 for all mammals; concave curvature for β_2_ < 0).

The four equations ((1), (2), (3), (4)) relating log_10_ BMR to log_10_ BM were basal to our four statistical scenarios. In the first scenario, we used ordinary least squares regression analysis (OLS) to evaluate equations (1) through (4). The respective scaling models applied to orders, marsupials, eutherians, and all mammals are denoted L, L_0.75_, L_0.67_, and C. These assume that neither a correction for their shared evolutionary history nor for differences in *T* values of species improves the metabolic scaling relationship of a given mammalian taxon. In the second scenario, we used again OLS, but additionally corrected for *T*. We therefore introduced the term β_3_/*T* (Kolokotrones et al., 2010) into equations (1) through (4). This resulted in four additional equations and in the four scaling models L_T_, L_0.75,T_, L_0.67,T_, and C_T_. In the third and fourth scenarios, we applied phylogenetic informed regression analysis (PGLS; Pagel, 1997, 1999; Freckleton et al., 2002) instead of OLS to control for a shared evolutionary history of species. Thus, scaling models L_PGLS_, L_0.75,PGLS_, L_0.67,PGLS_, and C_PGLS_ using equations (1) through (4) corrected only for phylogeny, whereas L_T,PGLS_, L_0.75,T,PGLS_, L_0.67,T,PGLS_, and C_T,PGLS_ using equations (1) through (4) with the temperature term corrected for both phylogeny and differences in *T* values between species. In total, we considered for each of the 17 orders, Marsupialia, Eutheria, and all mammals four statistical scenarios (OLS without and with temperature correction, PGLS without and with temperature correction) and four equations. This resulted in a total of 16 scaling models tested for each mammalian taxon studied.

For each order, marsupials, eutherians, and all mammals, we identified the overall statistical best out of the 16 models considered by two approaches. First, to rate absolute goodness of fit of models obtained for each of the analyzed taxa we always inspected their residual standard errors. Second, to assess relative goodness of fit of models, we used the corrected Akaike information criterion (AICc; Burnham & Anderson, 2002) values. We preferred AICc over standard AIC values, because sample sizes of some of the studied orders were small (≥5, Figure 1; Burnham & Anderson, 2002). For large sample sizes, there is nearly no difference between AICc and AIC values. We identified the best of the candidate models for a given taxon by model selection and followed the AIC evaluation approach given in Burnham and Anderson (2002). Therefore, at first, all candidate models were ranked according to their AICc values, and then the statistically best supported model with the lowest AICc (min(AICc)) was identified. Next, ∆AICc values (AICc‐min(AICc)) were calculated for each of all other candidate models. A ∆AICc score less than two suggests well‐supported models, a score between two and ten suggests a moderate support, and a score larger than ten suggests a weak support of the model relative to the alternative model (with the lowest AICc; Burnham & Anderson, 2002).

When a linear model worked best in terms of AICc for a given order, the marsupials, eutherians, or all mammals, we inspected the model's slope. If it had a fixed slope, this either indicated a statistical support of a ¾ or a ⅔ power scaling. If the model estimating slopes and intercepts (L, L_T_, L_PGLS_, or L_T,PGLS_) worked best, we checked, whether 0.75 and 0.67 is found in the 95% confidence interval of the slope to assess which scaling exponent(s) is (are) statistically supported. If several models obtained a similar statistical support (∆AICc ≤ 2) for a given taxon, we merged the information on the exponents provided by each of these models.

In our last analysis, we examined correlations between all beta coefficients estimated by models L, L_T_, L_PGLS_, and L_T,PGLS_ across orders. We therefore conducted Spearman rank correlation analysis for pairs of coefficients obtained for all orders studied (slope β_1_ vs. intercept β_0_, slope β_1_ vs. temperature coefficient β_3_, intercept β_0_ vs. temperature coefficient β_3_). With this analysis, we extended the empirical study of Sieg et al. (2009) on correlations between slopes and intercepts when examining heterogeneous taxonomic levels of mammals to scaling models accounting for differences in species’ body temperatures. With this analysis, we aimed to assess whether temperature informed models are able to disentangle the effect of BM, *T*, and other factors on BMR.

All statistical analyses were carried out in the free statistics software R (version 3.0.2). For curve fittings, we used the packages *nlme* (version 3.1.111) and *ape* (version 3.1.4).

## 3 Results

**Figure 2:**
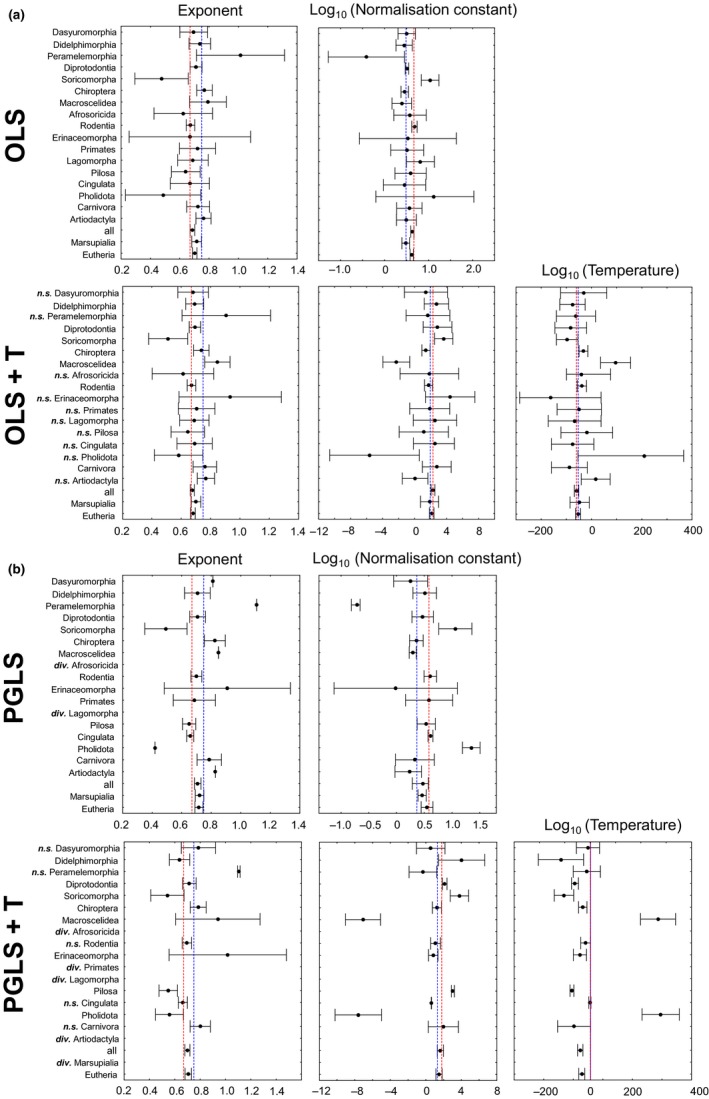
Metabolic scaling in 17 mammalian orders, Marsupialia, Eutheria, and all mammals. Shown are beta coefficients of linear models estimated under four statistical scenarios (L, L_T_, L_PGLS_, L_T__,_ _PGLS_), and their 95% confidence intervals (whiskers). In all panels, the marsupialian and eutherian orders are separated, and marsupialian and eutherian orders are ordered by the average body masses of taxa (median, S21 in the Supporting information). Exact values of regression models are found in Tables S1 through S20 in the Supporting Information. Confidence intervals of estimated slopes, intercepts, and if applicable of the coefficients of the temperature term of models L, L_T_, L_PGLS_, and L_T__,_ _PGLS_ are also given in the Supporting Information. (a) OLS: ordinary least squares regression analysis without correction for temperature and phylogeny, OLS + T.: OLS with correction for temperature, but not for phylogeny, (b) PGLS: phylogenetic generalized least squares regression analysis (Pagel 1997, 1999; Freckleton et al., 2002) without correction for temperature, PGLS + T: PGLS with correction for temperature. Slope panels: red lines = ⅔ power scaling, blue lines = ¾ power scaling. Intercept panels: red lines = intercept of the all mammals model with a fixed slope of 0.67, blue lines = intercept of the all mammals model with a fixed slope of 0.75. Temperature panels: red lines = temperature coefficient of the all mammals model with a fixed slope of 0.67, blue lines = temperature coefficient of the all mammals model with a fixed slope of 0.75. * = all beta coefficients (slope, intercept, temperature) differ significantly from zero, *n.s*. = at least one of the coefficients does not significantly differ from zero, *div*. = no model could be established, the fitting algorithm diverged

Figure 2 summarizes models L, L_T_, L_PGLS_, and L_T,PGLS_ obtained for orders, marsupials, eutherians, and all mammals graphically. Exact values of the beta coefficients of all linear and curvilinear regression models tested, their residual standard errors, and their AICc values are found in Tables S1 through S20 in the Supporting Information. Table 1 lists ∆AICc values of all 16 models considered for each of the mammalian taxa studied and provides information on scaling exponents corroborated by best models.

**Table 1:** ΔAICc values derived for scaling models and statistical scenarios studied for different mammalian taxa. Statistical scenarios and respective scaling models are OLS (L, L_0.75_, L_0.67_, C), OLS+T (L_T_, L_0.75,T_, L_0.67,T_, C_T_), PGLS (L_PGLS_, L_0.75,PGLS_, L_0.67,PGLS_, C_PGLS_), and PGLS+T (L_T,PGLS_, L_0.75,T,PGLS_, L_0.67,T,PGLS_, C_T,PGLS_). Straight line denotes models L, L_T_, L_PGLS_, and L_T,PGLS_, respectively, straight line β_1_ = 0.75 denotes models L_0.75_, L_0.75,T_, L_0.75,PGLS_, and L_0.75,T,PGLS_, respectively, and straight line β_1_ = 0.67 denotes models L_0.67_, L_0.67,T_, L_0.67,PGLS_, and L_0.67,T,PGLS_, respectively. The overall best models in terms of the lowest AICc values out of the 16 models tested is marked in bold for each taxon (all models differing in their ΔAICc not more than 2 compared with the model with the lowest AICc, Burnham & Anderson, 2002). For linear models, it is shown which information on the exponent is supported by the bold models for a taxon. For quadratic models, the shape of the curvature is given, that is, whether it is convex (β_2_ > 0) or concave (β_2_ < 0). *N* = number of species analyzed; *n.s*.: nonsignificant model, *div*: no convergence of fitting, *inf*.: infinity. The complete statistics of models and their regression coefficients are found in Tables S1 through S20 in the Supporting Information

Taxon	*N*	Statistical scenario	Straight line	Straight line β_1_ = 0.75	Straight line β_1_ = 0.67	Exponent	Quadratic	Shape
*All mammals*	519	OLS	214.5	279.4	217.0		205.0	Convex
		OLS+T	59.7	175.1	58.1		37.0	Convex
		PGLS	37.8	48.1	47.8		198.0	Convex
		PGLS+T	**0**	21.8	4.6	**(0.67, 0.75)**	32.9	Convex
*Marsupialia*	63	OLS	27.1	29.1	31.9		*n.s*.	
		OLS+T	23.6	*n.s*.	24.3		*n.s*.	
		PGLS	**0**	14.6	34.0	**(0.67, 0.75]**	*div*.	
		PGLS+T	*div*.	*div*.	26.5		*div*.	
*Eutheria*	456	OLS	154.8	184.8	166.6		139.1	Convex
		OLS+T	61.4	132.6	61.0		34.7	Convex
		PGLS	22.6	26.2	32.8		127.6	Convex
		PGLS+T	**0**	8.9	6.0	**(0.67, 0.75)**	*div*.	
Dasyuromorphia	21	OLS	19.2	17.8	16.7		*n.s*.	
		OLS+T	*n.s*.	*n.s*.	*n.s*.		*n.s*.	
		PGLS	3.4	14.2	18.1		*div*.	
		PGLS+T	*n.s*.	6.0	**0**	**= 0.67**	*div*.	
Didelphimorphia	11	OLS	7.4	3.7	7.0		*n.s*.	
		OLS+T	4.6	*n.s*.	**0**	**= 0.67**	*n.s*.	
		PGLS	11.9	7.6	5.7		10.7	Concave
		PGLS+T	16.7	*div*.	2.3		*n.s*.	
Peramelemorphia	8	OLS	16.7	14.4	16.0		12.8	Concave
		OLS+T	*n.s*.	*n.s*.	*n.s*.		*n.s*.	.
		PGLS	**0**	5.5	14.1	**>0.75**	23.4	Concave
		PGLS+T	*n.s*.	19.0	*div*.		77.1	Concave
Diprotodontia	23	OLS	26.4	27.4	27.7		*n.s*.	
		OLS+T	22.8	*n.s*.	21.4		*n.s*.	
		PGLS	24.9	24.3	24.0		*n.s*.	
		PGLS+T	3.2	**0**	2.6	**= 0.75**	*n.s*.	
Soricomorpha	23	OLS	23.7	29.1	25.4		*n.s*.	
		OLS+T	10.8	19.0	13.4		*n.s*.	
		PGLS	21.2	28.2	23.2		*n.s*.	
		PGLS+T	**0**	*div*.	*div*.	**≤0.67**	*div*.	
Chiroptera	73	OLS	13.8	12.1	24.0		*n.s*.	
		OLS+T	3.5	**1.6**	7.6	**= 0.75**	*n.s*.	
		PGLS	9.5	11.2	24.1		*n.s*.	
		PGLS+T	**1.3**	**0**	*div*.	**(0.67, 0.75]**	*n.s*.	
Macroscelidea	8	OLS	7.0	**2.0**	5.3	**= 0.75**	*n.s*.	
		OLS+T	7.7	*n.s*.	*n.s*.		*n.s*.	
		PGLS	**0**	7.6	10.5	**>0.75**	*n.s*.	
		PGLS+T	8.8	*n.s*.	**0.5**	**= 0.67**	*n.s*.	
Afrosoricida	9	OLS	4.0	**1.5**	**0**	**= 0.75, = 0.67**	*n.s*.	
		OLS+T	*n.s*.	*n.s*.	*n.s*.		*n.s*.	
		PGLS	*div*.	5.5	3.3	= 0.67	*div*.	
		PGLS+T	*div*.	5.2	2.9		*div*.	
Rodentia	236	OLS	44.4	66.9	42.4		42.4	Convex
		OLS+T	29.7	55.9	27.6		27.7	Convex
		PGLS	**1.2**	5.6	2.2	**[0.67, 0.75)**	36.5	Convex
		PGLS+T	*n.s*.	*n.s*.	**0**	**= 0.67**	25.2	Convex
Erinaceomorpha	7	OLS	14.1	7.3	7.1		*n.s*.	
		OLS+T	*n.s*.	*n.s*.	*n.s*.		*n.s*.	
		PGLS	24.7	11.2	11.7		*div*.	
		PGLS+T	41.6	**0**	**1.6**	**= 0.75, = 0.67**	*n.s*.	
Primates	18	OLS	2.6	**0**	**0.5**	**= 0.75, = 0.67**	*n.s*.	
		OLS+T	*n.s*.	*n.s*.	*n.s*.		*n.s*.	
		PGLS	5.8	4.4	3.8		*div*.	
		PGLS+T	*div*.	*div*.	*div*.		*div*.	
Lagomorpha	11	OLS	13.0	10.5	9.2		*n.s*.	
		OLS+T	*n.s*.	*n.s*.	*n.s*.		*n.s*.	
		PGLS	*div*.	**0**	*div*.	**= 0.75**	*n.s*.	
		PGLS+T	*div*.	*div*.	*div*.		*n.s*.	
Pilosa	6	OLS	9.2	7.4	3.3		*n.s*.	
		OLS+T	*n.s*.	*n.s*.	*n.s*.		*n.s*.	
		PGLS	8.4	10.7	**0**	**= 0.67**	*+inf*.	
		PGLS+T	*inf*.	*div*.	*n.s*.		*n.s*.	
Cingulata	9	OLS	17.8	14.6	13.0		18.5	Concave
		OLS+T	*n.s*.	*n.s*.	*n.s*		*n.s*.	
		PGLS	5.9	*n.s*.	**0**	**= 0.67**	23.6	Concave
		PGLS+T	*n.s*.	*n.s*.	*n.s*.		*n.s*.	
Pholidota	5	OLS	122.6	*n.s*.	105.1		*n.s*.	
		OLS+T	*n.s*.	*n.s*.	*n.s*.		**0**	**Concave**
		PGLS	*inf*.	98.2	109.9		31.4	Concave
		PGLS+T	177.8	*inf*.	*inf*.		185.8	Concave
Carnivora	43	OLS	10.2	8.3	9.7		**1.0**	**Convex**
		OLS+T	6.7	4.4	*n.s*.		*n.s*.	
		PGLS	**1.7**	**0**	6.4	**(0.67, 0.75]**	*div*.	
		PGLS+T	*n.s*.	*n.s*.	*n.s*.		*div*.	
Artiodactyla	8	OLS	27.1	21.7	30.4		*n.s*.	
		OLS+T	*n.s*.	*n.s*.	*n.s*.		*n.s*.	
		PGLS	29.1	**0**	20.2	**= 0.75**	*div*.	
		PGLS+T	*div*.	*div*.	*div*.		*div*.	

### 3.1 Best models scaling models for mammalian taxa studied

Except for Pholidota and Carnivora, a linear model worked best in terms of AICc for all mammalian taxa studied. In Pholidota, the concave (β_2_ < 0) curvilinear scaling relationship worked best and in Carnivora the second best model (∆AICc ≤ 2) was a convex relationship (β_2_ > 0). In terms of residual standard error, a concave curvature was best for Pholidota and Peramelemorphia, and a convex one for Carnivora, Rodentia, Eutheria, and all mammals (Table S1 through S20 in the Supporting Information). For the other 14 taxa studied, the model with the lowest residual standard error had also the lowest AICc value.

### 3.2 ¾ or ⅔ power scaling or none of both in mammalian taxa studied

For Diprotodontia, Lagomorpha, and Artiodactyla, a linear model with a fixed slope of 0.75 was best in terms of AICc, which indicates a scaling exponent of ¾ in these orders. For Dasyuromorphia, Didelphimorphia, Pilosa, and Cingulata, models with a slope fixed to 0.67 were best in terms of AICc values suggesting a ⅔ power scaling for these orders. For all mammals, marsupials, eutherians, Peramelemorphia, and Soricomorpha, models estimating the slope and intercept obtained the highest support in terms of AICc values. The 95% confidence intervals of estimated slopes suggested an exponent intermediary to ⅔ and ¾ for all mammals and eutherians, larger than ⅔ but not than ¾ for marsupials, larger than ¾ for Peramelemorphia, and smaller than or equal to ⅔ for Soricomorpha. For Chiroptera, Macroscelidea, Afrosoricida, Rodentia, Erinaceomorpha, and Primates, more than one linear model obtained a similar support in terms of AICc values (∆AICc ≤ 2). For Chiroptera, these indicated an exponent larger than ⅔ but not larger than ¾, for Macroscelidea ⅔, ¾ or even larger, for Afrosoricida and Erinaceomorpha ⅔ or ¾, for Rodentia ⅔ but smaller than ¾, and for Primates ⅔ up to ¾. For Pholidota, for which a concave curvature obtained clearly the highest support in terms of AICc the best linear model suggested an exponent of ¾. For Carnivora, two linear models and a convex curvature obtained a similar high support in terms of AICc. The two linear models indicated an exponent larger than ⅔ but not larger than ¾ in Carnivora. In total, we observed a high variability in scaling exponents of the 20 analyzed mammalian taxa and found no support for a single scaling exponent across all mammals and also none for a single exponent in marsupilian and eutherian orders.

### 3.3 Correlations between coefficients of models L, L_T_, L_PGLS_, and L_T,PGLS_

**Figure 3:**
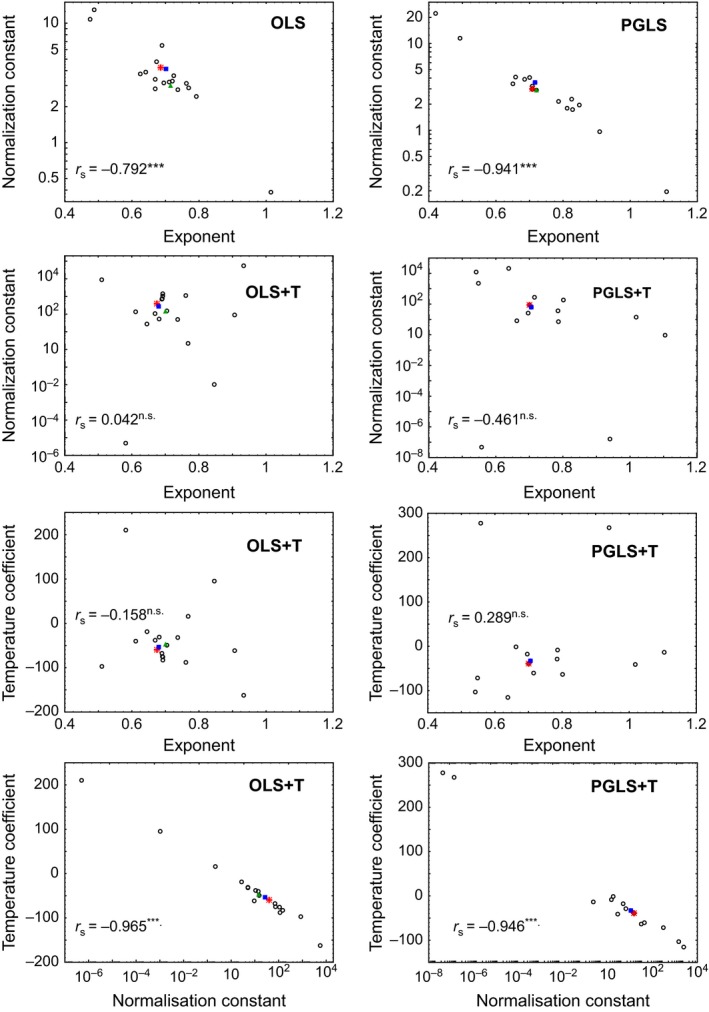
Relationships between beta coefficients of models L, L_T_, L_PGLS_, and L_T__,_ _PGLS_ for orders studied. The correlations between coefficients of orders were assessed by Spearman rank correlation analyses (*r*_s_; n.s. *p *> .05, ***p < .001). Red asterisk: all mammals, green triangle: Marsupialia, blue square: Eutheria. Exact values of regression coefficients (normalization constant, exponent, temperature coefficient) are found in Tables S1 through S20 in the Supporting Information.

Exponents (β_1_) and normalization constants (10^β_0_) of models L and L_PGLS_ estimated for orders correlated highly negatively (Figure 3). Higher exponents resulted in lower normalization constants and vice versa. When correcting for temperature (L_T,_ L_T,PGLS_), exponents and normalization constants of orders were uncorrelated, and also exponents and temperature term coefficients (β_3_). Contrary, normalization constants and values of the coefficient of the temperature term correlated highly negatively (Figure 3).

### 3.4 Correction for a shared evolutionary history and body temperature of species needed?

For seven of a total of 20 mammalian taxa analyzed, a model correcting for body temperature and the shared evolutionary history of species clearly worked best in terms of AICc. These were all mammals, the Eutheria, the two marsupilian orders Dasyuromorphia and Diprotodontia, and the three eutherian orders Soricomorpha, Chiroptera, and Erinaceomorpha. For seven taxa, the Marsupialia, the marsupilian order Peramelemorphia, and the eutherian orders Lagomorpha, Pilosa, Cingulata, Carnivora, and Artiodactyla, the best model corrected only for phylogeny, and for the marsupilian order Didelphimorphia and the eutherian order Pholidota, it corrected only for differences in body temperatures between species. For the eutherians Macroscelidea, three best models in terms of AICc (∆AICc ≤ 2) were identified. The one with the highest statistical support corrected only for phylogeny, that with the second highest support corrected for phylogeny and temperature, and that with the lowest support for none of both. For the eutherians Rodentia, the best model corrected for both body temperature and phylogeny and the second best only for phylogeny (Table 1). In total, whether correction for differences in body temperature between species and for their shared evolutionary history improved the scaling model obtained depended on the mammalian taxon studied.

## 4 Discussion

### 4.1 Best scaling models of mammalian taxa

A clear statistical support of a curvilinear scaling relationship in terms of AIC values and residual standard errors was only observed for Pholidota. However, our dataset on Pholidota is the smallest of this study (*N* = 5), especially in comparison with the number of model parameters estimated. This strongly questions whether Pholidota in general show a concave curvilinear scaling relationship. For all other orders, marsupilians, eutherians, and all mammals, the model with the lowest AICc value was linear (Table 1). Our results thus strongly question a curvilinear scaling in mammals, marsupials, eutherians, and in at least 16 of 17 orders studied. For all mammals, this observation is unexpected as numerous papers applying different statistical approaches report a slight, convex curvature of BMR versus BM (Capellini et al., 2010; Clarke et al., 2010; Isaac & Carbone, 2010; Kolokotrones et al., 2010; Müller et al., 2012). A statistical problem in detecting a potential curvature in all marsupilian orders, except for Diprotodontia and in all eutherian orders, except for Rodentia and Carnivora could be related to their small body mass range covered. Müller et al. (2012) showed that this range should comprise four up to five magnitudes in order to find a slight curvature in mammals.

However, when using residual standard errors to select the best model for a mammalian taxon, a convex curvature worked best for all mammals, but also for Eutheria, Peramelemorphia, Carnivora, and Rodentia. For Carnivora, the second best model in terms of AICc values was also a convex curvature. For all mammals, the difference in AICc values between the best linear model and the best curvilinear model was 32.9, which is a considerable stronger support of the linear over the quadratic model than indicated by their small difference in residual standard errors (0.145 vs. 0.185, Table S1 in the Supporting Information).

Whether the curvilinear scaling of mammalian metabolism is a true pattern is highly discussed. Deficiencies of the analyzed datasets themselves and statistical problems are reasons for this (e.g., MacKay, 2011; Müller et al., 2012; Packard, 2015). Body masses, metabolic rates, and temperatures of species are often sampled from multiple sources (Packard, 2015) or body mass ranges are not broad enough to resemble the slight curvature (Müller et al., 2012). Kolokotrones et al. (2010) applied *R* ^2^ for model selection, which is incorrect when comparing linear and nonlinear models (Quinn & Keough, 2002). For the total dataset of Sieg et al. (2009), they observed an increase in *R* ^2^ from 0.958 (linear model) to 0.961 (curvature), which is anyway minimal. As the curvature in BMR strongly questions the theoretical framework of the MTE and led to a modification of the WBE by Kolokotrones et al. (2010), other studies aimed at whether the curvature in metabolic scaling is also seen in other taxa and whether it scales up to higher ecological levels. For the more ecologically relevant field metabolic rate (FMR), Hudson, Isaac, and Reuman (2013) and Bueno and López‐Urrutia (2014) corroborated a curvilinear scaling in mammals, but for birds, Hudson et al. (2013) found no statistical support for a curvature in FMR. For mammals, a curvature in BMR and FMR was also demonstrated by Müller et al. (2012), but these authors found no curvature in the scaling of BMR for reptiles and birds. Bueno and López‐Urrutia (2014) showed that the curvature seen in BMR and FMR prevailed in six further mammalian traits (four individual traits and two population traits), but their scaling coefficients derived were not consistent with those expected from the MTE. All these results suggest that curvilinear scaling relationships are a phenomenon only seen in mammals. They strongly question the WBE modification done by Kolokotrones et al. (2010), especially because the convex curvature is a slight pattern.

In our study, only residual standard errors indicated curvilinear scaling relationships in Peramelemorphia, Carnivora, and Rodentia. Hayssen and Lacy (1985) also found a convex curvature for Carnivora, whereas Rodentia showed a linear scaling. Clarke et al. (2010) detected a convex curvature for both Carnivora and Rodentia, and none in any other mammalian order (with a sample size >20), but these authors did not study the Peramelemorphia. Several other authors have studied Rodentia and Carnivora (Capellini et al., 2010; Duncan et al., 2007; Sieg et al., 2009; White & Seymour, 2004). They detected differences in the exponents between these orders and found a high variability even within each of the orders. The latter could reflect different ecological characteristics of species (McNab, 2008) and could yield spurious results on the presence and absence of a curvature in these orders. However, in all orders for which a curvature was only indicated by residual standard errors in our study, the difference between the curvilinear and best linear model was small (difference is 0.061 for Peramelemorphia, 0.056 for Carnivora, 0.014 for Rodentia).

The scaling exponents of the 17 mammalian orders studied herein showed a high variability and thus corroborated no single exponent in mammals (Table 1). Even the orders from marsupials and eutherians had no single exponent (Table 1). When evaluating all best linear models for mammalian taxa studied (Table 1), the Soricomorpha had the lowest exponent. It was smaller than or equal to ⅔. A ⅔ power scaling was observed in Dasyuromorphia, Didelphimorphia, and Xenarthra (Cingulata, Pilosa). A ¾ power scaling was seen in Diprotodontia, Lagomorpha, Artiodactyla, and Pholidota. In Peramelemorphia, an even higher exponent than ¾ was indicated. For Afrosoricidea, Erinaceomorpha, and Primates, neither a ⅔ nor a ¾ power scaling was rejected. The Chiroptera and Carnivora could have an exponent higher than ⅔ but not greater than ¾, whereas the exponent of Rodentia could be ⅔ or smaller than ¾. For Macroscelidea, the exponent could be ⅔, ¾ or even higher. For all mammals and Eutheria, an exponent intermediary to ⅔ and ¾ was indicated, whereas in Marsupialia an exponent of ¾ was indicated. Thus, our results contradict previous findings that orders dominated by larger species have larger scaling exponents than orders dominated by smaller species (Figure 2; Glazier, 2005; Duncan et al., 2007; Clarke et al., 2010).

Other studies corroborate our results on the considerable variability found in exponents of mammalian orders (Table 2), although the statistical methods used in these studies are diverse, sample sizes and species composition for orders generally differ between studies, and even the taxonomic status of species could have changed in the meantime. For the comparison of our results with those of previous studies, we always chose our most similar statistical scenario as reference (Table 2). Overall, only in four cases the slopes of our reference models considerably differed from respective literature values (Table 2). In the majority of cases, our results matched literature values (20) or differences were only marginal (12, Table 2). The intermediary exponent seen under temperature and phylogenetic correction in all mammals matched the results of Sieg et al. (2009) and Clarke et al. (2010).

**Table 2:** Comparison of scaling exponents of orders derived in this study with those obtained by other authors. We only used literature that at least corrects for body temperature or for phylogeny and compared exponents to those obtained by us under the most similar statistical correction scenario (reference model). Source = author(s) of the study, Method = method(s) used by author(s). Ref. model = our reference model. #Orders = number of orders which are comparable. Orders different = order(s) for which literature results differ from ours. Orders matched = order(s) for which literature results are in agreement with ours. Cursive indicates orders for which only small differences exist between literature exponents and those obtained in this study. See column Notes for justification

Source	Method	Ref. model	#Orders	Orders different	Orders matched	Notes
Sieg et al. (2009)	PGLS analysis with temperature correction	L_T,PGLS_	2	No	Rodentia, Chiroptera	Predicted exponents of models L_T,PGLS_ matched those reported in Sieg et al. (2009), the authors applied the identical statistical approach.
Glazier (2005)	OLS with temperature correction	L_T_	4	No	Chiroptera, Dasyuromorphia, Rodentia, Afrosoricida	Glazier (2005) reports results from White and Seymour (2003)
White and Seymour (2004)	OLS analysis and normalization of BMRs to a body temperature of 36.2°C	L_T_	8	*Didelphimorphia, Chiroptera*	Carnivora, Dasyuromorphia, Diprotodontia, Primates, Rodentia, Xenarthra, *Didelphimorphia, Chiroptera*	Evaluation of 95% confidence intervals given in White and Seymour (2004). The authors can neither reject ¾ nor ⅔ for Didelphimorphia and Chiroptera, and our study suggests ⅔ for Didelphimorphia and ¾ for Chiroptera.
Duncan et al. (2007)	PGLS analysis	L_PGLS_	12	Dasyuromorphia, *Afrosoricida, Didelphimorphia, Lagomorpha, Pilosa, Cingulata, Artiodactyla*	Rodentia, Carnivora, Chiroptera, Diprotodontia, Primates*, Afrosoricida, Didelphimorphia, Lagomorpha, Pilosa, Cingulata, Artiodactyla*	Visual inspection of 95% confidence intervals of slopes shown in Figure 3 from Duncan et al. (2007). The authors studied Xenarthra as a whole, and our study predicts equal exponents for Pilosa and Cingulata. For Dasyuromorphia, the authors could neither reject ⅔ nor ¾, whereas model L_PGLS_ predicts an exponent larger than ¾. For Afrosoricida, the authors observed an exponent of ⅔ or even smaller, and this study could neither reject ⅔ nor ¾, for Didelphimorphia, the authors could neither reject ¾ nor ⅔, whereas this study suggests ¾, for Lagomorpha, Primates, Pilosa, Cingulata, and Artiodactyla, the authors could neither reject ⅔ nor ¾, whereas this study suggests ¾ for Lagomorpha and Artiodactyla and ⅔ for Pilosa and Cingulata.
Capellini et al. (2010)	Phylogenetically independent contrasts	L_PGLS_	3	Rodentia, *Chiroptera, Carnivora*	*Chiroptera, Carnivora*	Capellini et al. (2010) report ¾ or an even higher exponent for Rodentia, Chiroptera, and Carnivora, and this study suggests ¾ for Chiroptera and Carnivora, and ⅔ for Rodentia.
Clarke et al. (2010)	Phylogenetic independent contrasts and corrected for body temperature	L_T,PGLS_	6	Dasyuromorphia, Diprotodontia, *Soricomorpha*	Rodentia, Carnivora, Chiroptera, *Soricomorpha*	We used exponents and standard errors given by Clarke et al. (2010) for six orders in their Supplementary Material. For Dasyuromorphia, the authors reject ⅔ and showed that the exponent is ¾ or even higher, whereas our model L_T,PGLS_ suggests ⅔. For Diprotodontia, the authors found ⅔, whereas L_T,PGLS_ predicts ¾. For Soricomorpha, the authors could neither reject ⅔ nor ¾ and our model L_T,PGLS_ predicts ⅔.

### 4.2 Correlations between coefficients of models L, L_T_, L_PGLS_, and L_T,PGLS_

The mathematical interdependence of normalization constants and exponents in simple allometric relationships (*y *= *a x* ^*b*^) has not been sufficiently appreciated, but makes the biological interpretation of two allometric regression lines problematic, when their exponents differ (Gould, 1966; White & Gould, 1965). To the best of our knowledge, Sieg et al. (2009) were the only authors who empirically demonstrated this correlation in the last years. As expected (Gould, 1966; White & Gould, 1965) and consistent with Sieg et al. (2009), our estimated exponents (β_1_) and normalization constants (10^β_0_) of models L and L_PGLS_ derived for orders correlated strongly (Figure 3). This correlation was considerably stronger under PGLS than under OLS (Figure 3). PGLS, thus, captures an important source of variation that caused a stronger deviation from the mathematically expected correlation between the exponent and the normalization constant under OLS. This observation corroborates again that phylogenetic informed analysis improves scaling relationships (White, 2011). When correcting for body temperature (models L_T_ and L_T_,_PGLS_), the correlation between exponents (β_1_) and normalization constants (10^β_0_) disappeared, and also no correlation between the exponent and the respective temperature coefficients (β_3_) was seen (Figure 3). Thus, the introduction of the temperature term removed the mathematically expected correlation between the exponent and the normalization constant, and now the normalization constant and the temperature coefficient might only capture additional sources of variation in BMR besides BM. However, under temperature correction a strong correlation between the normalization constant (10^β_0_) and the temperature coefficient (β_3_) emerged (Figure 3). As the exponents and the temperature terms are uncorrelated, the temperature term and the normalization constant could reflect other factors besides body mass influencing metabolic scaling in mammalian orders. On the one hand, this would suggest that differences in body temperature of similar‐sized species are the most important factor driving differences in BMRs of similar‐sized species and that the Arrhenius approach used by the MTE (Brown et al., 2004) is corroborated. In this case, the small proportion of variability not explained by the temperature coefficient and captured by the normalization constant would reflect differences in other ecological characteristics of species (e.g., torpor, diet, habitat; McNab, 2008, 2012). On the other, the high correlation between the temperature coefficient and the normalization constant could indicate that differences in the ecology of species are linked to differences in body temperature (e.g., carnivores have higher body temperatures than herbivores) and that these are the most important factor driving differences in BMRs of similar‐sized species.

### 4.3 Correction for a shared evolutionary history and body temperature of species needed?

The majority of best models found for mammalian taxa studied by us corrected for a shared phylogeny of species. OLS models clearly worked best in terms of AICc only for Didelphimorphia, Afrosoricida, Primates, and Pholidota (Table 1), but we think only for Primates the support of the OLS over the PGLS model reflects a true pattern. Sample sizes of Didelphimorphia, Afrosoricida, and Pholidota were among the smallest of all taxa studied (Figure 1) and λ values estimated by the respective phylogenetically corrected models were considerable smaller or larger than expected from theory (0 ≤ λ ≤ 1, Pagel, 1997, 1999; Freckleton et al., 2002; Tables S5, S11, and S18 of the Supporting Information). Contrary, for Primates, the phylogenetic signal under PGLS analysis was low and λ was within the expected range (Table S14 of the Supporting Information). Our study thus corroborates that phylogenetic informed analysis improves scaling relationships obtained (White, 2011).

For ten of the 20 mammalian taxa studied, the best model in terms of AICc did not correct for differences in species’ body temperature (Table 1). For Didelphimorphia, Peramelemorphia, Afrosoricida, Primates, Lagomorpha, Pilosa, Cingulata, Carnivora, and Artiodactyla, this observation is corroborated by the absence of a correlation between BMR and *T* (Table 3), whereas for marsupials, not only BMR and *T*, but also BM and *T* correlated (Table 3). Thus, in marsupials the effect of *T* on BMR, which is not taken into consideration by the best model, could have been captured by BM. Contrary, in all mammals, eutherians, Dasyuromorphia, and Chiroptera, in which BMR and *T* as well as BM and *T* correlate (Table 3) the best model still corrected for *T* (Table 1). In these taxa, the correlation between BMR and *T* is considerable stronger than between BM and *T*, except for Dasyuromorphia (Table 3). In Dasyuromorphia, both correlations are similarly strong, which is consistent with the observation that the best model correcting for species’ body temperatures is only moderately supported over the model ignoring body temperature differences of species (Table 1). In Soricomorpha, the presence of the temperature term in the best scaling model (Table 2) is corroborated by the strong correlation between BMR and *T* (Table 3). In Diprotodontia, Macroscelidea, Erinaceomorpha, and Pholidota, the best model also corrected for *T* (Table 2), but the correlation analysis indicated that BMR and *T* are uncorrelated (Table 3). The latter could be due to the small sample sizes of orders which hampers the statistical detection of weaker significant correlations (Table 3), but also due to model overfitting. For Erinaceomorpha, even the correlation between BM and BMR was not significant. For Rodentia, the best model corrected for temperature, but the second best (∆AICc = 1.2) not, which is consistent with the absence of a correlation between BMR and *T* (Table 3).

**Table 3:** Results of Spearman rank correlation analysis on BM, BMR, and *T* for different mammalian taxa. *r*_s_ (BMR vs. *T*) assesses whether body temperatures of species affect their basal metabolic rate; that is, *T* could potentially account for differences in BMR of species in scaling models correcting for *T*,* r*_s_ (BM vs. *T*) whether *T* increases or decreases with species body mass or BM has no effect on *T*, and *r*_s_ (BM vs. BMR) whether BM influences BMR. Significant *r*_s_ values (*p *≤ .05) are marked in bold

Taxon	*N*	*r*_s_ (BMR vs. *T*)	*p*	*r*_s_ (BM vs. *T*)	*p*	*r*_s_ (BM vs. BMR)	*p*
*All mammals*	519	**0.231**	<10^−7^	**0.129**	0.003	**0.962**	<10^−15^
*Marsupialia*	63	**0.461**	<10^−4^	**0.432**	<10^−3^	**0.955**	<10^−15^
*Eutheria*	456	**0.346**	<10^−13^	**0.252**	<10^−7^	**0.955**	<10^−15^
Dasyuromorphia	21	**0.533**	0.013	**0.582**	0.006	**0.952**	<10^−5^
Didelphimorphia	11	0.532	0.092	0.532	0.092	**1.000**	<10^−15^
Peramelemorphia	8	0.398	0.329	0.096	0.820	**0.833**	0.015
Diprotodontia	23	0.239	0.273	0.144	0.513	**0.979**	<10^−5^
Soricomorpha	23	**0.414**	0.049	**−**0.141	0.521	**0.681**	<10^−4^
Chiroptera	73	**0.373**	0.001	**0.288**	0.013	**0.935**	<10^−15^
Macroscelidea	8	0.096	0.820	0.428	0.291	**0.867**	0.005
Afrosoricida	9	0.091	0.802	0.018	0.960	**0.939**	<10^−15^
Rodentia	236	0.074	0.253	**−**0.027	0.673	**0.902**	<10^−15^
Erinaceomorpha	7	**−**0.180	0.699	**−**0.234	0.613	0.750	0.067
Primates	18	0.322	0.179	0.230	0.345	**0.946**	<10^−5^
Lagomorpha	11	0.009	0.989	**−**0.164	0.634	**0.818**	0.004
Pilosa	6	0.232	0.658	**−**0.203	0.700	0.600	0.242
Cingulata	9	**−**0.126	0.748	**−**0.159	0.683	**0.917**	0.001
Pholidota	5	0.205	0.741	0.205	0.741	**1.000**	0.017
Carnivora	43	**−**0.226	0.145	**−0.386**	0.011	**0.930**	<10^−15^
Artiodactyla	8	0.323	0.435	0.323	0.435	**1.000**	<10^−5^

## 5 Conclusions

Our analyses corroborate previous studies that reject a single scaling exponent in mammalian taxa (Capellini et al., 2010; Clarke et al., 2010; Duncan et al., 2007; Isaac & Carbone, 2010; McNab, 2008; Müller et al., 2012; Sieg et al., 2009; White & Seymour, 2004). They corroborate that phylogenetic informed analysis improves scaling relationships obtained (White, 2011). Correcting for differences in species’ body temperatures removed the mathematically built‐in correlation between the scaling exponent and the normalization constant of standard scaling relationships (*y *= *a x* ^*b*^; White & Gould, 1965; Gould, 1966), but the biological interpretation of the normalization constant and the temperature coefficient is still problematic as both strongly correlate at least in the studied mammalian taxa. In 14 of 20 taxa studied BMR and *T* did not correlate, which questions the need of a temperature correction for these taxa. In six taxa, *T* correlated with BM positively or negatively. This hampers a disentangling of the effect of BM and *T* on BMR, and an interpretation of how *T* and other factors besides BM influence the scaling of BMR in any mammalian taxon.

## Conflict of Interest

None declared.

## Data Accessibility

The dataset used in this study is published by Sieg et al. (2009) and available in Dryad (http://hdl.handle.net/10255/dryad.713).

All other data used in this manuscript are present in the manuscript and its Supporting Information.
