# Supplementary material for: Mass, phylogeny, and temperature are sufficient to explain differences in metabolic scaling across mammalian orders?
Source: Ecol Evol. 2016 Oct 24;6(23):8352–65. doi: 10.1002/ece3.2555 (PMC5167101; doi:10.1002/ece3.2555)
Supplement: Supplementary file 1 [file ECE3-6-8352-s001.docx]

**Supporting Information**

Table S1. Metabolic scaling in all mammals. Data set on mammals is a subset from Sieg *et al.* (2009). It comprises those species for which temperature and phylogenetic information is available. Estimated beta values (ß.), their 95% confidence intervals (95%CI), their p-values, residual standard errors of models (Res.Se.), AICc and ΔAIC values of models are listed for all 16 models studied. ß_0_ = intercept, ß_1_ = slope, ß_2_ = coefficient of the quadratic term, ß_3_ = coefficient of the temperature term, λ = strength of the phylogenetic signal (only PGLS).

| model | N | *ß_0_* | 95%CI | p | *ß_1_* | 95%CI | p | *ß_2_* | 95%CI | p | *ß_3_* | 95%CI | p | λ | Res.S.E. | AICc | ΔAIC |
| --- | --- | --- | --- | --- | --- | --- | --- | --- | --- | --- | --- | --- | --- | --- | --- | --- | --- |
| L | 519 | 0.629 | 0.594, 0.664 | <10^-16^ | 0.686 | 0.670, 0.702 | <10^-16^ |  |  |  |  |  |  |  | 0.171 | -368.2 | 214.5 |
| C | 519 | 0.753 | 0.673, 0.754 | <10^-16^ | 0.571 | 0.502, 0.640 | <10^-16^ | 0.023 | 0.009, 0.037 | <10^-3^ |  |  |  |  | 0.169 | -377.7 | 205.0 |
| L_0.75_ | 519 | 0.487 | 0.471, 0.503 | <10^-16^ |  |  |  |  |  |  |  |  |  |  | 0.182 | -303.3 | 279.4 |
| L_0.67_ | 519 | 0.665 | 0.641, 0.678 | <10^-16^ |  |  |  |  |  |  |  |  |  |  | 0.171 | -365.7 | 217.0 |
| L_T_ | 519 | 2.292 | 2.047, 2.537 | <10^-16^ | 0.675 | 0.661, 0.689 | <10^-16^ |  |  |  | -59.277 | -67.909, -50.645 | <10^-16^ |  | 0.148 | -523.0 | 59.7 |
| C_T_ | 519 | 2.493 | 2,242, 2.744 | <10^-16^ | 0.529 | 0.470, 0.588 | <10^-16^ | 0.028 | 0.016, 0.040 | <10^-6^ | -60.876 | -69.341, -52.411 | <10^-16^ |  | 0.145 | -545.7 | 37.0 |
| L_0.75,T_ | 519 | 1.943 | 1.678, 2.208 | <10^-16^ |  |  |  |  |  |  | -52.706 | -62.247, -43.165 | <10^-16^ |  | 0.165 | -407.6 | 175.1 |
| L_0.67,T_ | 519 | 2.313 | 2.076, 2.434 | <10^-16^ |  |  |  |  |  |  | -59.669 | -68.222, -51.116 | <10^-16^ |  | 0.148 | -524.6 | 58.1 |
| L_PGLS_ | 519 | 0.478 | 0.282, 0.574 | <10^-5^ | 0.709 | 0.687, 0.731 | <10^-16^ |  |  |  |  |  |  | 0.772 | 0.225 | -544.9 | 37.8 |
| C_PGLS_ | 519 | 0.753 | 0.643, 0.862 | <10^-16^ | 0.578 | 0.509, 0.712 | <10^-16^ | 0.021 | 0.007, 0.035 | 0.001 |  |  |  | 0.167 | 0.176 | -384.7 | 198.0 |
| L_0.75,PGLS_ | 519 | 0.364 | 0.162, 0.566 | <10^-3^ |  |  |  |  |  |  |  |  |  | 0.809 | 0.239 | -534.6 | 48.1 |
| L_0.67,PGLS_ | 519 | 0.587 | 0.403, 0.771 | <10^-9^ |  |  |  |  |  |  |  |  |  | 0.759 | 0.224 | -534.9 | 47.8 |
| L_T,PGLS_ | 519 | 1.645 | 1.294, 1.996 | <10^-16^ | 0.700 | 0.680, 0.720 | <10^-16^ |  |  |  | -38.900 | -49.658, -28.142 | <10^-11^ | 0.606 | 0.185 | -582.7 | **0** |
| C_T,PGLS_ | 519 | 2.472 | 2.219, 2.725 | <10^-16^ | 0.530 | 0.471, 0.589 | <10^-16^ | 0.028 | 0.016, 0.040 | <10^-6^ | -60.300 | -68.728, -51.872 | <10^-16^ | 0.060 | 0.145 | -549.8 | 32.9 |
| L_0.75,T,PGLS_ | 519 | 1.306 | 0.938, 1.674 | <10^-11^ |  |  |  |  |  |  | -32.365 | -43.557, -21.173 | <10^-7^ | 0.730 | 0.212 | -560.9 | 21.8 |
| L_0.67,T,PGLS_ | 519 | 1.790 | 1.453, 2.127 | <10^-16^ |  |  |  |  |  |  | -41.423 | -52.095, -30.751 | <10^-13^ | 0.571 | 0.181 | -578.1 | 4.6 |

Table S2. Metabolic scaling in Marsupialia. Data set on Marsupialia is a subset from Sieg *et al.* (2009). It comprises those species for which temperature and phylogenetic information is available. Estimated beta values (ß.), their 95% confidence intervals (95%CI), their p-values, residual standard errors of models (Res.Se.), AICc and ΔAIC values of models are listed for all 16 models studied. ß_0_ = intercept, ß_1_ = slope, ß_2_ = coefficient of the quadratic term, ß_3_ = coefficient of the temperature term, λ = strength of the phylogenetic signal (only PGLS). *n.s*.: non-significant model, *div*: no convergence of fitting, *inf.*: infinity.

| model | N | *ß_0_* | 95%CI | p | *ß_1_* | 95%CI | p | *ß_2_* | 95%CI | p | *ß_3_* | 95%CI | p | λ | Res.S.E. | AICc | ΔAIC |
| --- | --- | --- | --- | --- | --- | --- | --- | --- | --- | --- | --- | --- | --- | --- | --- | --- | --- |
| L | 63 | 0.479 | 0.391, 0.567 | <10^-14^ | 0.715 | 0.682, 0.748 | <10^-16^ |  |  |  |  |  |  |  | 0.128 | -75.5 | 27.1 |
| C | 63 | 0.508 | 0.310, 0.706 | < 10^-10^ | 0.688 | 0.519, 0.857 | < 10^-10^ | 0.006 | -0.025, 0.037 | 0.746 |  |  |  |  | 0.129 | -73.3 | *n.s.* |
| L_0.75_ | 63 | 0.391 | 0.358, 0.424 | < 10^-16^ |  |  |  |  |  |  |  |  |  |  | 0.132 | -73.5 | 29.1 |
| L_0.67_ | 63 | 0.592 | 0.559, 0.625 | < 10^-16^ |  |  |  |  |  |  |  |  |  |  | 0.135 | -70.7 | 31.9 |
| L_T_ | 63 | 1.870 | 0.735, 3.003 | 0.002 | 0.700 | 0.667, 0.733 | < 10^-16^ |  |  |  | -47.691 | -86.519, -8.863 | 0.019 |  | 0.123 | -79.0 | 23.6 |
| C_T_ | 63 | 1.900 | 0.743, 3.056 | 0.002 | 0.671 | 0.508, 0.834 | < 10^-10^ | 0.006 | -0.025, 0.037 | 0.727 | -47.728 | -86.843, -8.612 | 0.020 |  | 0.125 | -76.8 | *n.s.* |
| L_0.75,T_ | 63 | 1.148 | 0.066, 2.230 | 0.042 |  |  |  |  |  |  | -26.737 | -64.943, 11.469 | 0.175 |  | 0.131 | -73.2 | *n.s.* |
| L_0.67,T_ | 63 | 2.295 | 1.256, 3.334 | < 10^-4^ |  |  |  |  |  |  | -60.140 | -96.817, -23.463 | 0.002 |  | 0.126 | -78.3 | 24.3 |
| L_PGLS_ | 63 | 0.461 | 0.387, 0.535 | < 10^-16^ | 0.721 | 0.692, 0.750 | < 10^-16^ |  |  |  |  |  |  | -0.051 | 0.128 | -102.6 | **0** |
| C_PGLS_ | 63 |  |  |  |  |  |  |  |  |  |  |  |  |  |  |  | *div.* |
| L_0.75,PGLS_ | 63 | 0.391 | 0.391, 0.391 | < 10^-16^ |  |  |  |  |  |  |  |  |  | -0.051 | 0.130 | -88.0 | 14.6 |
| L_0.67,PGLS_ | 63 | 0.594 | 0.543, 0.645 | < 10^-16^ |  |  |  |  |  |  |  |  |  | 0.087 | 0.135 | -68.9 | 34.0 |
| L_T,PGLS_ | 63 |  |  |  |  |  |  |  |  |  |  |  |  |  |  |  | *div.* |
| C_T,PGLS_ | 63 |  |  |  |  |  |  |  |  |  |  |  |  |  |  |  | *div.* |
| L_0.75,T,PGLS_ | 63 |  |  |  |  |  |  |  |  |  |  |  |  |  |  |  | *div.* |
| L_0.67,T,PGLS_ | 63 | 2.294 | 1.247, 3.341 | < 10^-4^ |  |  |  |  |  |  | -60.021 | -96.955, -23087 | 0.002 | 0.020 | 0.126 | -76.1 | 26.5 |

Table S3. Metabolic scaling in Eutheria. Data set on Eutheria is a subset from Sieg *et al.* (2009). It comprises those species for which temperature and phylogenetic information is available. Estimated beta values (ß.), their 95% confidence intervals (95%CI), their p-values, residual standard errors of models (Res.Se.), AICc and ΔAIC values of models are listed for all 16 models studied. ß_0_ = intercept, ß_1_ = slope, ß_2_ = coefficient of the quadratic term, ß_3_ = coefficient of the temperature term, λ = strength of the phylogenetic signal (only PGLS). *n.s*.: non-significant model, *div*: no convergence of fitting, *inf.*: infinity.

| model | N | *ß_0_* | 95%CI | p | *ß_1_* | 95%CI | p | *ß_2_* | 95%CI | p | *ß_3_* | 95%CI | p | λ | Res.S.E. | AICc | ΔAIC |
| --- | --- | --- | --- | --- | --- | --- | --- | --- | --- | --- | --- | --- | --- | --- | --- | --- | --- |
| L | 456 | 0.616 | 0.577, 0.653 | < 10^-16^ | 0.702 | 0.686, 0.717 | < 10^-16^ |  |  |  |  |  |  |  | 0.168 | -324.6 | 154.8 |
| C | 456 | 0.776 | 0.694, 0.858 | < 10^-16^ | 0.551 | 0.478, 0.623 | < 10^-16^ | 0.029 | 0.015, 0.043 | < 10^-16^ |  |  |  |  | 0.164 | -340.3 | 139.1 |
| L_0.75_ | 456 | 0.513 | 0.497, 0.529 | < 10^-16^ |  |  |  |  |  |  |  |  |  |  | 0.173 | -294.6 | 184.8 |
| L_0.67_ | 456 | 0.683 | 0.667, 0.699 | < 10^-16^ |  |  |  |  |  |  |  |  |  |  | 0.170 | -312.8 | 166.6 |
| L_T_ | 456 | 2.127 | 1.836, 2.417 | < 10^-16^ | 0.680 | 0.664, 0.696 | < 10^-16^ |  |  |  | -53.445 | -63.635, -43.255 | < 10^-16^ |  | 0.151 | -418.0 | 61.4 |
| C_T_ | 456 | 2.357 | 2.065, 2.649 | < 10^-16^ | 0.508 | 0.443, 0.573 | < 10^-16^ | 0.034 | 0.022, 0.046 | < 10^-16^ | -55.111 | -65.009, 65.009 | < 10^-16^ |  | 0.146 | -444.7 | 34.7 |
| L_0.75,T_ | 456 | 1.643 | 1.351, 1.935 | < 10^-16^ |  |  |  |  |  |  | -41.196 | -51.849, -30.543 | < 10^-16^ |  | 0.163 | -346.8 | 132.6 |
| L_0.67,T_ | 456 | 2.197 | 1.927, 2.467 | < 10^-16^ |  |  |  |  |  |  | -55.210 | -65.0433, -45.377 | < 10^-16^ |  | 0.151 | -418.4 | 61.0 |
| L_PGLS_ | 456 | 0.550 | 0.444, 0.656 | < 10^-16^ | 0.717 | 0.690, 0.744 | < 10^-16^ |  |  |  |  |  |  | 0.690 | 0.191 | -456.8 | 22.6 |
| C_PGLS_ | 456 | 0.800 | 0.712, 0.888 | < 10^-16^ | 0.539 | 0.468, 0.610 | < 10^-16^ | 0.031 | 0.017, 0.045 | < 10^-16^ |  |  |  | 0.132 | 0.165 | -351.8 | 127.6 |
| L_0.75,PGLS_ | 456 | 0.475 | 0.383, 0.567 | < 10^-16^ |  |  |  |  |  |  |  |  |  | 0.717 | 0.196 | -453.2 | 26.2 |
| L_0.67,PGLS_ | 456 | 0.659 | 0.571, 0.747 | < 10^-16^ |  |  |  |  |  |  |  |  |  | 0.679 | 0.192 | -446.6 | 32.8 |
| L_T,PGLS_ | 456 | 1.492 | 1.143, 1.841 | < 10^-16^ | 0.706 | 0.681, 0.731 | < 10^-16^ |  |  |  | -32.830 | -44.715, -20.945 | < 10^-16^ | 0.577 | 0.170 | -479.4 | **0** |
| C_T,PGLS_ | 456 |  |  |  |  |  |  |  |  |  |  |  |  |  |  |  | *div.* |
| L_0.75,T,PGLS_ | 456 | 1.269 | 0.924, 1.614 | < 10^-16^ |  |  |  |  |  |  | -28.474 | -40.544, -16.404 | < 10^-16^ | 0.637 | 0.181 | -470.5 | 8.9 |
| L_0.67,T,PGLS_ | 456 | 1.658 | 1.321, 1.995 | < 10^-16^ |  |  |  |  |  |  | -35.793 | -47.629, -29.755 | < 10^-16^ | 0.522 | 0.168 | -473.4 | 6.0 |

Table S4. Metabolic scaling in Dasyuromorphia. Data set on Dasyuromorphia is a subset from Sieg *et al.* (2009). It comprises those species for which temperature and phylogenetic information is available. Estimated beta values (ß.), their 95% confidence intervals (95%CI), their p-values, residual standard errors of models (Res.Se.), AICc and ΔAIC values of models are listed for all 16 models studied. ß_0_ = intercept, ß_1_ = slope, ß_2_ = coefficient of the quadratic term, ß_3_ = coefficient of the temperature term, λ = strength of the phylogenetic signal (only PGLS). *n.s*.: non-significant model, *div*: no convergence of fitting, *inf.*: infinity.

| model | N | *ß_0_* | 95%CI | p | *ß_1_* | 95%CI | p | *ß_2_* | 95%CI | p | *ß_3_* | 95%CI | p | λ | Res.S.E. | AICc | ΔAIC |
| --- | --- | --- | --- | --- | --- | --- | --- | --- | --- | --- | --- | --- | --- | --- | --- | --- | --- |
| L | 21 | 0.501 | 0.303, 0.699 | <10^-4^ | 0.695 | 0.601, 0.789 | <10^-11^ |  |  |  |  |  |  |  | 0.182 | -6.6 | 19.2 |
| C | 21 | 0.544 | -0.005, 0.814 | 0.064 | 0.649 | 0.100, 1.198 | 0.032 | 0.010 | -0.096, 0.133 | 0.870 |  |  |  |  | 0.187 | -3.6 | *n.s.* |
| L_0.75_ | 21 | 0.395 | 0.317, 0.473 | <10^-8^ |  |  |  |  |  |  |  |  |  |  | 0.184 | -8.0 | 17.8 |
| L_0.67_ | 21 | 0.549 | 0.473, 0.625 | <10^-11^ |  |  |  |  |  |  |  |  |  |  | 0.179 | -9.1 | 16.7 |
| L_T_ | 21 | 1.411 | -1.276, 4.098 | 0.317 | 0.681 | 0.577, 0.785 | <10^-9^ |  |  |  | -31.127 | -122.824, 60.570 | 0.514 |  | 0.185 | -4.0 | *n.s.* |
| C_T_ | 21 | 1.495 | -1.367, 4.357 | 0.320 | 0.619 | 0.055, 1.183 | 0.046 | 0.014 | -0.111, 0.139 | 0.828 | -32.051 | -126.631, 62.529 | 0.515 |  | 0.190 | -0.6 | *n.s.* |
| L_0.75,T_ | 21 | 0.614 | -1.832, 0.560 | 0.628 |  |  |  |  |  |  | -7.704 | -93.830, 78.422 | 0.863 |  | 0.188 | -5.3 | *n.s.* |
| L_0.67,T_ | 21 | 1.542 | -0.800, 3.884 | 0.212 |  |  |  |  |  |  | -34.990 | -117.449, 47.469 | 0.416 |  | 0.180 | -7.1 | *n.s.* |
| L_PGLS_ | 21 | 0.254 | -0.052, 0.560 | 0.121 | 0.812 | 0.812, 0.812 | <10^-16^ |  |  |  |  |  |  | 1.083 | 0.298 | -22.4 | 3.4 |
| C_PGLS_ | 21 |  |  |  |  |  |  |  |  |  |  |  |  |  |  |  | *div.* |
| L_0.75,PGLS_ | 21 | 0.367 | 0.063, 0.671 | 0.028 |  |  |  |  |  |  |  |  |  | 1.083 | 0.296 | -11.6 | 14.2 |
| L_0.67,PGLS_ | 21 | 0.512 | 0.191, 0.833 | 0.005 |  |  |  |  |  |  |  |  |  | 1.083 | 0.312 | -7.7 | 18.1 |
| L_T,PGLS_ | 21 | 0.550 | -1.057, 2.157 | 0.510 | 0.787 | 0.652, 0.922 | <10^-9^ |  |  |  | -8.596 | -54.276, 37.084 | 0.717 | 1.083 | 0.305 | -24.7 | *n.s.* |
| C_T,PGLS_ | 21 |  |  |  |  |  |  |  |  |  |  |  |  |  |  |  | *div.* |
| L_0.75,T,PGLS_ | 21 | 0.974 | 0.666, 1.282 | <10^-5^ |  |  |  |  |  |  | -20.903 | -21.115, -20.691 | <10^-16^ | 1.083 | 0.299 | -19.8 | 6.0 |
| L_0.67,T,PGLS_ | 21 | 1.901 | 1.574, 2.228 | <10^-9^ |  |  |  |  |  |  | -47.777 | -47.779, -47.775 | <10^-16^ | 1.083 | 0.319 | -25.8 | **0** |

Table S5. Metabolic scaling in Didelphimorphia. Data set on Didelphimorphia is a subset from Sieg *et al.* (2009). It comprises those species for which temperature and phylogenetic information is available. Estimated beta values (ß.), their 95% confidence intervals (95%CI), their p-values, residual standard errors of models (Res.Se.), AICc and ΔAIC values of models are listed for all 16 models studied. ß_0_ = intercept, ß_1_ = slope, ß_2_ = coefficient of the quadratic term, ß_3_ = coefficient of the temperature term, λ = strength of the phylogenetic signal (only PGLS). *n.s*.: non-significant model, *div*: no convergence of fitting, *inf.*: infinity.

| Model | N | *ß_0_* | 95%CI | p | *ß_1_* | 95%CI | p | *ß_2_* | 95%CI | p | *ß_3_* | 95%CI | p | λ | Res.S.E. | AICc | ΔAIC |
| --- | --- | --- | --- | --- | --- | --- | --- | --- | --- | --- | --- | --- | --- | --- | --- | --- | --- |
| L | 11 | 0.443 | 0.257, 0.629 | 0.001 | 0.737 | 0.664, 0.810 | <10^-8^ |  |  |  |  |  |  |  | 0.077 | -18.0 | 7.4 |
| C | 11 | 0.785 | 0.252, 1.318 | 0.020 | 0.417 | -0.059, 0.893 | 0.220 | 0.069 | -0.033, 0.171 | 0.220 |  |  |  |  | 0.073 | -14.9 | *n.s.* |
| L_0.75_ | 11 | 0.412 | 0.369, 0.455 | <10^-8^ |  |  |  |  |  |  |  |  |  |  | 0.074 | -21.7 | 3.7 |
| L_0.67_ | 11 | 0.612 | 0.561, 0.663 | <10^-9^ |  |  |  |  |  |  |  |  |  |  | 0.086 | -18.4 | 7.0 |
| L_T_ | 11 | 2.732 | 1.201, 4.263 | 0.008 | 0.692 | 0.631, 0.753 | <10^-7^ |  |  |  | -75.447 | -125.729, -25.165 | 0.019 |  | 0.057 | -20.8 | 4.6 |
| C_T_ | 11 | 2.666 | 1.074, 4.257 | 0.013 | 0.552 | 0.158, 0.946 | 0.028 | 0.031 | -0.033, 0.089 | 0.505 | -68.245 | -123.940, -12.550 | 0.047 |  | 0.059 | -14.2 | *n.s.* |
| L_0.75,T_ | 11 | 1.903 | 0.480, 3.326 | 0.028 |  |  |  |  |  |  | -51.732 | -101.075, 2.389 | 0.070 |  | 0.064 | -22.0 | *n.s.* |
| L_0.67,T_ | 11 | 3.040 | 1.817, 4.263 | 0.001 |  |  |  |  |  |  | -84.256 | -126.661, -41.851 | 0.004 |  | 0.055 | -25.4 | **0** |
| L_PGLS_ | 11 | 0.509 | 0.297, 0.721 | 0.001 | 0.708 | 0.620, 0.793 | <10^-7^ |  |  |  |  |  |  | 0.764 | 0.082 | -13.5 | 11.9 |
| C_PGLS_ | 11 | -1.739 | -2.227, -1.251 | <10^-4^ | 2.429 | 2.221, 2.638 | <10^-7^ | -0.307 | -0.338, -0.276 | <10^-7^ |  |  |  | 1.434 | 0.394 | -14.7 | 10.7 |
| L_0.75,PGLS_ | 11 | 0.412 | 0.351, 0.473 | <10^-6^ |  |  |  |  |  |  |  |  |  | 0.475 | 0.077 | -17.8 | 7.6 |
| L_0.67,PGLS_ | 11 | 0.646 | 0.581, 0.711 | <10^-8^ |  |  |  |  |  |  |  |  |  | 2.294 | 0.067 | -19.7 | 5.7 |
| L_T,PGLS_ | 11 | 4.036 | 1.429, 6.643 | 0.016 | 0.638 | 0.558, 0.718 | <10^-6^ |  |  |  | -115.293 | -204.765, -25.821 | 0.035 | 1.434 | 0.079 | -8.7 | 16.7 |
| C_T,PGLS_ | 11 | 3.162 | 1.568, 4.756 | 0.006 | 0.882 | 0.545, 1.219 | 0.001 | -0.055 | -0.126, 0.016 | 0.165 | -94.003 | -148.328, -39.678 | 0.012 | 1.434 | 0.075 | -0.2 | *n.s.* |
| L_0.75,T,PGLS_ | 11 |  |  |  |  |  |  |  |  |  |  |  |  |  |  |  | *div.* |
| L_0.67,T,PGLS_ | 11 | 3.442 | 1.813, 5.071 | 0.003 |  |  |  |  |  |  | -96.872 | -152.975, -40.769 | 0.008 | 1.709 | 0.048 | -23.1 | 2.3 |

Table S6. Metabolic scaling in Peramelemorphia. Data set on Peramelemorphia is a subset from Sieg *et al.* (2009). It comprises those species for which temperature and phylogenetic information is available. Estimated beta values (ß.), their 95% confidence intervals (95%CI), their p-values, residual standard errors of models (Res.Se.), AICc and ΔAIC values of models are listed for all 16 models studied. ß_0_ = intercept, ß_1_ = slope, ß_2_ = coefficient of the quadratic term, ß_3_ = coefficient of the temperature term, λ = strength of the phylogenetic signal (only PGLS). *n.s*.: non-significant model, *div*: no convergence of fitting, *inf.*: infinity.

| Model | N | *ß_0_* | 95%CI | p | *ß_1_* | 95%CI | p | *ß_2_* | 95%CI | p | *ß_3_* | 95%CI | p | λ | Res.S.E. | AICc | ΔAIC |
| --- | --- | --- | --- | --- | --- | --- | --- | --- | --- | --- | --- | --- | --- | --- | --- | --- | --- |
| L | 8 | -0.414 | -1.282, 0.454 | 0.387 | 1.014 | 0.714, 1.314 | 0.001 |  |  |  |  |  |  |  | 0.071 | -10.0 | 16.7 |
| C | 8 | -14.208 | -20.100, -8.316 | 0.005 | 10.878 | 6.674, 15.082 | 0.004 | -1.755 | -2.504, -1.006 | 0.006 |  |  |  |  | 0.034 | -13.9 | 12.8 |
| L_0.75_ | 8 | 0.351 | 0.296, 0.406 | <10^-5^ |  |  |  |  |  |  |  |  |  |  | 0.080 | -12.3 | 14.4 |
| L_0.67_ | 8 | 0.583 | 0.522, 0.644 | <10^-6^ |  |  |  |  |  |  |  |  |  |  | 0.089 | -10.7 | 16.0 |
| L_T_ | 8 | 1.663 | -1.065, 4.391 | 0.286 | 0.907 | 0.605, 1.209 | 0.002 |  |  |  | -61.830 | -139.667, 16.007 | 0.180 |  | 0.064 | -3.8 | *n.s.* |
| C_T_ | 8 | -11.562 | -17.532, -5.592 | 0.019 | 9.701 | 5.818, 13.584 | 0.008 | -1.556 | -2.242, -0.870 | 0.011 | -32.115 | -70.170, 5.940 | 0.173 |  | 0.029 | 0.6 | *n.s.* |
| L_0.75,T_ | 8 | 2.637 | 0.639, 4.634 | 0.041 |  |  |  |  |  |  | -79.993 | -149.891, 10.095 | 0.066 |  | 0.064 | -11.6 | *n.s.* |
| L_0.67,T_ | 8 | 3.133 | 0.926, 5.340 | 0.032 |  |  |  |  |  |  | -89.240 | -166.480, 12.001 | 0.064 |  | 0.071 | -10.0 | *n.s.* |
| L_PGLS_ | 8 | -0.711 | -0.817, -0.657 | <10^-5^ | 1.108 | 1.108, 1.108 | <10^-6^ |  |  |  |  |  |  | 1.085 | 0.085 | -26.7 | **0** |
| C_PGLS_ | 8 | -13.282 | -19.378, -7.186 | 0.008 | 10.174 | 5.782, 14.566 | 0.006 | -1.623 | -2.411, -0.835 | 0.001 |  |  |  | -0.326 | 0.030 | -3.3 | 23.4 |
| L_0.75,PGLS_ | 8 | 0.347 | 0.347, 0.347 | <10^-16^ |  |  |  |  |  |  |  |  |  | -0.327 | 0.073 | -21.2 | 5.5 |
| L_0.67,PGLS_ | 8 | 0.592 | 0.592, 0.592 | <10^-16^ |  |  |  |  |  |  |  |  |  | -1.996 | 0.056 | -12.6 | 14.1 |
| L_T,PGLS_ | 8 | -0.326 | -1.896, 1.244 | 0.701 | 1.105 | 1.095, 1.115 | <10^-10^ |  |  |  | -13.231 | -67.094, 40.632 | 0.651 | 1.058 | 0.091 | -5.9 | *n.s.* |
| C_T,PGLS_ | 8 | -9.899 | -13.617, -6.181 | 0.006 | 8.645 | 6.219, 11.071 | 0.002 | -1.367 | -1.802, -0.932 | 0.004 | -38.523 | -60.941, -16.105 | 0.028 | 1.120 | 0.024 | 50.4 | 77.1 |
| L_0.75,T,PGLS_ | 8 | 3.740 | 1.343, 6.137 | 0.022 |  |  |  |  |  |  | -118.958 | -202.730, -35.186 | 0.032 | -0.566 | 0.054 | -7.7 | 19.0 |
| L_0.67,T,PGLS_ | 8 |  |  |  |  |  |  |  |  |  |  |  |  |  |  |  | *div.* |

Table S7. Metabolic scaling in Diprotodontia. Data set on Diprotodontia is a subset from Sieg *et al.* (2009). It comprises those species for which temperature and phylogenetic information is available. Estimated beta values (ß.), their 95% confidence intervals (95%CI), their p-values, residual standard errors of models (Res.Se.), AICc and ΔAIC values of models are listed for all 16 models studied. ß_0_ = intercept, ß_1_ = slope, ß_2_ = coefficient of the quadratic term, ß_3_ = coefficient of the temperature term, λ = strength of the phylogenetic signal (only PGLS). *n.s*.: non-significant model, *div*: no convergence of fitting, *inf.*: infinity.

| model | N | *ß_0_* | 95%CI | p | *ß_1_* | 95%CI | p | *ß_2_* | 95%CI | p | *ß_3_* | 95%CI | p | λ | Res.S.E. | AICc | ΔAIC |
| --- | --- | --- | --- | --- | --- | --- | --- | --- | --- | --- | --- | --- | --- | --- | --- | --- | --- |
| L | 23 | 0.507 | 0.381, 0.548 | <10^-7^ | 0.711 | 0.670, 0.752 | <10^-16^ |  |  |  |  |  |  |  | 0.107 | -32.2 | 26.4 |
| C | 23 | 0.464 | 0.188, 0.740 | <10^-3^ | 0.750 | 0.523, 0.975 | <10^-6^ | -0.004 | -0.048, 0.037 | 0.735 |  |  |  |  | 0.110 | -29.3 | *n.s.* |
| L_0.75_ | 23 | 0.392 | 0.347, 0.437 | <10^-13^ |  |  |  |  |  |  |  |  |  |  | 0.114 | -31.2 | 27.4 |
| L_0.67_ | 23 | 0.626 | 0.579, 0.673 | <10^-16^ |  |  |  |  |  |  |  |  |  |  | 0.114 | -30.9 | 27.7 |
| L_T_ | 23 | 2.862 | 1.063, 4.661 | 0.005 | 0.693 | 0.654, 0.732 | <10^-16^ |  |  |  | -82.457 | -145.306, -19.608 | 0.018 |  | 0.095 | -35.8 | 22.8 |
| C_T_ | 23 | 3.192 | 1.110, 5.274 | 0.007 | 0.621 | 0.400, 0.843 | <10^-4^ | 0.013 | -0.026, 0.052 | 0.527 | -91.293 | -160.499, -22.087 | 0.018 |  | 0.097 | -33.0 | *n.s.* |
| L_0.75,T_ | 23 | 1.770 | -0.141, 3.681 | 0.084 |  |  |  |  |  |  | -49.334 | -117.760, 19.092 | 0.172 |  | 0.111 | -30.6 | *n.s.* |
| L_0.67,T_ | 23 | 3.298 | 1.642, 4.954 | 0.001 |  |  |  |  |  |  | -95.665 | -154.910, -36.420 | 0.005 |  | 0.096 | -37.2 | 21.4 |
| L_PGLS_ | 23 | 0.471 | 0.277, 0.665 | <10^-4^ | 0.708 | 0.655, 0.761 | <10^-16^ |  |  |  |  |  |  | 0.973 | 0.130 | -33.7 | 24.9 |
| C_PGLS_ | 23 | 0.430 | 0.161, 0.699 | 0.005 | 0.756 | 0.538, 0.974 | <10^-5^ | -0.007 | -0.048, 0.034 | 0.737 |  |  |  | 0.309 | 0.111 | -26.9 | *n.s.* |
| L_0.75,PGLS_ | 23 | 0.345 | 0.223, 0.467 | <10^-5^ |  |  |  |  |  |  |  |  |  | 1.019 | 0.139 | -34.3 | 24.3 |
| L_0.67,PGLS_ | 23 | 0.586 | 0.462, 0.692 | <10^-10^ |  |  |  |  |  |  |  |  |  | 0.900 | 0.126 | -34.6 | 24.0 |
| L_T,PGLS_ | 23 | 2.128 | 1.869, 2.387 | <10^-12^ | 0.715 | 0.662, 0.768 | <10^-16^ |  |  |  | -59.932 | -73.389, -46.475 | <10^-7^ | 1.115 | 0.151 | -55.4 | 3.2 |
| C_T,PGLS_ | 23 | 3.077 | 1.248, 4.906 | 0.004 | 0.621 | 0.413, 0.829 | <10^-5^ | 0.015 | -0.022, 0.052 | 0.451 | -88.427 | -148.885, -27.969 | 0.010 | 0.395 | 0.097 | -31.5 | *n.s.* |
| L_0.75,T,PGLS_ | 23 | 2.275 | 2.136, 2.414 | <10^-16^ |  |  |  |  |  |  | -68.971 | -68.971, -68.971 | <10^-16^ | 1.115 | 0.154 | -58.6 | **0** |
| L_0.67,T,PGLS_ | 23 | 1.940 | 1.800, 2.083 | <10^-16^ |  |  |  |  |  |  | -48.458 | -48.459, -48.457 | <10^-16^ | 1.115 | 0.158 | -56.0 | 2.6 |

Table S8. Metabolic scaling in Soricomorpha. Data set on Soricomorpha is a subset from Sieg *et al.* (2009). It comprises those species for which temperature and phylogenetic information is available. Estimated beta values (ß.), their 95% confidence intervals (95%CI), their p-values, residual standard errors of models (Res.Se.), AICc and ΔAIC values of models are listed for all 16 models studied. ß_0_ = intercept, ß_1_ = slope, ß_2_ = coefficient of the quadratic term, ß_3_ = coefficient of the temperature term. ß_0_ = intercept, ß_1_ = slope, ß_2_ = coefficient of the quadratic term, ß_3_ = coefficient of the temperature term, λ = strength of the phylogenetic signal (only PGLS). *n.s*.: non-significant model, *div*: no convergence of fitting, *inf.*: infinity.

| model | N | *ß_0_* | 95%CI | p | *ß_1_* | 95%CI | p | *ß_2_* | 95%CI | p | *ß_3_* | 95%CI | p | λ | Res.S.E. | AICc | ΔAIC |
| --- | --- | --- | --- | --- | --- | --- | --- | --- | --- | --- | --- | --- | --- | --- | --- | --- | --- |
| L | 23 | 1.034 | 0.834, 1.234 | <10^-8^ | 0.476 | 0.294, 0.658 | <10^-4^ |  |  |  |  |  |  |  | 0.158 | -14.6 | 23.7 |
| C | 23 | 1.408 | 0.930, 1.886 | <10^-5^ | -0.305 | -1.233, 0.625 | 0.528 | 0.362 | -0.061, 0.785 | 0.109 |  |  |  |  | 0.151 | -14.6 | *n.s.* |
| L_0.75_ | 23 | 0.750 | 0..676, 0.824 | <10^-14^ |  |  |  |  |  |  |  |  |  |  | 0.183 | -9.2 | 29.1 |
| L_0.67_ | 23 | 0.833 | 0.764, 0.902 | <10^-16^ |  |  |  |  |  |  |  |  |  |  | 0.169 | -12.9 | 25.4 |
| L_T_ | 23 | 3.637 | 2.485, 4.789 | <10^-5^ | 0.511 | 0.378, 0.644 | <10^-6^ |  |  |  | -96.799 | -139.319, -54.279 | <10^-3^ |  | 0.114 | -27.5 | 10.8 |
| C_T_ | 23 | 3.676 | 2.535, 4.517 | <10^-5^ | 0.074 | -0.643, 0.791 | 0.842 | 0.202 | -0.123, 0.527 | 0.239 | -90.487 | -133.729, -47.245 | <10^-3^ |  | 0.113 | -25.9 | *n.s.* |
| L_0.75,T_ | 23 | 3.631 | 2.198, 5.064 | <10^-4^ |  |  |  |  |  |  | -105.656 | -158.141, -53.171 | <10^-3^ |  | 0.142 | -19.3 | 19.0 |
| L_0.67,T_ | 23 | 3.633 | 2.363, 4.903 | <10^-5^ |  |  |  |  |  |  | -102.688 | -149.224, -56.152 | <10^-3^ |  | 0.126 | -24.9 | 13.4 |
| L_PGLS_ | 23 | 1.060 | 0.762, 1.358 | <10^-6^ | 0.493 | 0.350, 0.636 | <10^-5^ |  |  |  |  |  |  | 0.996 | 0.228 | -17.1 | 21.2 |
| C_PGLS_ | 23 | 1.257 | 0.806, 1.708 | <10^-4^ | 0.132 | -0.728, 0.992 | 0.766 | 0.181 | -0.219, 0.581 | 0.384 |  |  |  | 0.737 | 0.168 | -17.8 | *n.s.* |
| L_0.75,PGLS_ | 23 | 0.758 | 0.546, 0.970 | <10^-6^ |  |  |  |  |  |  |  |  |  | 0.762 | 0.217 | -10.1 | 28.2 |
| L_0.67,PGLS_ | 23 | 0.852 | 0.640, 1.064 | <10^-7^ |  |  |  |  |  |  |  |  |  | 0.839 | 0.210 | -15.1 | 23.2 |
| L_T,PGLS_ | 23 | 3.795 | 2.754, 4.836 | <10^-6^ | 0.542 | 0.411, 0.673 | <10^-7^ |  |  |  | -103.556 | -141.992, -65.120 | <10^-4^ | -0.100 | 0.108 | -38.3 | **0** |
| C_T,PGLS_ | 23 |  |  |  |  |  |  |  |  |  |  |  |  |  |  |  | *div.* |
| L_0.75,T,PGLS_ | 23 |  |  |  |  |  |  |  |  |  |  |  |  |  |  |  | *div.* |
| L_0.67,T,PGLS_ | 23 |  |  |  |  |  |  |  |  |  |  |  |  |  |  |  | *div.* |

Table S9. Metabolic scaling in Chiroptera. Data set on Chiroptera is a subset from Sieg *et al.* (2009). It comprises those species for which temperature and phylogenetic information is available. Estimated beta values (ß.), their 95% confidence intervals (95%CI), their p-values, residual standard errors of models (Res.Se.), AICc and ΔAIC values of models are listed for all 16 models studied. ß_0_ = intercept, ß_1_ = slope, ß_2_ = coefficient of the quadratic term, ß_3_ = coefficient of the temperature term, λ = strength of the phylogenetic signal (only PGLS). *n.s*.: non-significant model, *div*: no convergence of fitting, *inf.*: infinity.

| model | N | *ß_0_* | 95%CI | p | *ß_1_* | 95%CI | p | *ß_2_* | 95%CI | p | *ß_3_* | 95%CI | p | λ | Res.S.E. | AICc | ΔAIC |
| --- | --- | --- | --- | --- | --- | --- | --- | --- | --- | --- | --- | --- | --- | --- | --- | --- | --- |
| L | 73 | 0.457 | 0.373, 0.541 | <10^-16^ | 0.769 | 0.716, 0.822 | <10^-16^ |  |  |  |  |  |  |  | 0.129 | -87.2 | 13.8 |
| C | 73 | 0.331 | 0.086, 0.576 | 0.010 | 0.936 | 0.628, 1.244 | <10^-7^ | -0.047 | -0.133, 0.260 | 0.284 |  |  |  |  | 0.129 | -86.2 | *n.s.* |
| L_0.75_ | 73 | 0.485 | 0.456, 0.514 | <10^-16^ |  |  |  |  |  |  |  |  |  |  | 0.129 | -88.9 | 12.1 |
| L_0.67_ | 73 | 0.603 | 0.572, 0.634 | <10^-16^ |  |  |  |  |  |  |  |  |  |  | 0.140 | -77.0 | 24.0 |
| L_T_ | 73 | 1.410 | 0.889, 1.931 | <10^-6^ | 0.737 | 0.684, 0.790 | <10^-16^ |  |  |  | -31.799 | -48.998, -14.600 | <10^-3^ |  | 0.119 | -97.5 | 3.5 |
| C_T_ | 73 | 1.285 | 0.721, 1.849 | <10^-4^ | 0.897 | 0.613, 1.181 | <10^-7^ | -0.046 | -0.126, 0.035 | 0.267 | -31.662 | -48.832, -14.492 | <10^-3^ |  | 0.119 | -96.5 | *n.s.* |
| L_0.75,T_ | 73 | 1.353 | 0.890, 1.816 | <10^-6^ |  |  |  |  |  |  | -30.441 | -46.614, -14.267 | <10^-3^ |  | 0.119 | -99.5 | 1.6 |
| L_0.67,T_ | 73 | 1.716 | 1.234, 2.198 | <10^-9^ |  |  |  |  |  |  | -39.035 | -55.907, -22.163 | <10^-4^ |  | 0.124 | -93.4 | 7.6 |
| L_PGLS_ | 73 | 0.357 | 0.235, 0.479 | <10^-6^ | 0.825 | 0.754, 0.896 | <10^-16^ |  |  |  |  |  |  | 0.467 | 0.139 | -91.5 | 9.5 |
| C_PGLS_ | 73 | 0.322 | 0.077, 0.567 | 0.012 | 0.949 | 0.641, 1.255 | <10^-7^ | -0.051 | -0.137, 0.035 | 0.246 |  |  |  | 0.042 |  |  | *n.s.* |
| L_0.75,PGLS_ | 73 | 0.463 | 0.400, 0.526 | <10^-16^ |  |  |  |  |  |  |  |  |  | 0.282 | 0.133 | -89.8 | 11.2 |
| L_0.67,PGLS_ | 73 | 0.573 | 0.502, 0.644 | <10^-16^ |  |  |  |  |  |  |  |  |  | 0.308 | 0.147 | -76.9 | 24.1 |
| L_T,PGLS_ | 73 | 1.247 | 0.737, 1.757 | <10^-5^ | 0.785 | 0.722, 0.848 | <10^-16^ |  |  |  | -28.937 | -45.628, -12.246 | 0.001 | 0.241 | 0.121 | -99.7 | 1.3 |
| C_T,PGLS_ | 73 | 1.377 | 0.832, 1.922 | <10^-5^ | 0.869 | 0.589, 1.149 | <10^-7^ | -0.040 | -0.120, 0.040 | 0.334 | -34.214 | -51.013, -17.415 | <10^-3^ | -0.034 | 0.120 | -94.5 | *n.s.* |
| L_0.75,T,PGLS_ | 73 | 1.371 | 0.905, 1.837 | <10^-6^ |  |  |  |  |  |  | -31.520 | -47.678, -15.362 | <10^-3^ | 0.165 | 0.119 | -101.0 | **0** |
| L_0.67,PGLS_ | 73 |  |  |  |  |  |  |  |  |  |  |  |  |  |  |  | *div.* |

Table S10. Metabolic scaling in Macroscelidea. Data set on Macroscelidea is a subset from Sieg *et al.* (2009). It comprises those species for which temperature and phylogenetic information is available. Estimated beta values (ß.), their 95% confidence intervals (95%CI), their p-values, residual standard errors of models (Res.Se.), AICc and ΔAIC values of models are listed for all 16 models studied. ß_0_ = intercept, ß_1_ = slope, ß_2_ = coefficient of the quadratic term, ß_3_ = coefficient of the temperature term, λ = strength of the phylogenetic signal (only PGLS). *n.s*.: non-significant model, *div*: no convergence of fitting, *inf.*: infinity.

| model | N | *ß_0_* | 95%CI | p | *ß_1_* | 95%CI | p | *ß_2_* | 95%CI | p | *ß_3_* | 95%CI | p | λ | Res.S.E. | AICc | ΔAIC |
| --- | --- | --- | --- | --- | --- | --- | --- | --- | --- | --- | --- | --- | --- | --- | --- | --- | --- |
| L | 8 | 0.388 | 0.165, 0.611 | 0.014 | 0.792 | 0.667, 0.917 | <10^-5^ |  |  |  |  |  |  |  | 0.039 | -19.6 | 7.0 |
| C | 8 | 1.548 | -1.216, 4.312 | 0.322 | -0.411 | -3.271, 2.449 | 0.789 | 0.305 | -0.293, 1.030 | 0.447 |  |  |  |  | 0.040 | -11.2 | *n.s.* |
| L_0.75_ | 8 | 0.463 | 0.438, 0.488 | <10^-8^ |  |  |  |  |  |  |  |  |  |  | 0.037 | -24.6 | 2.0 |
| L_0.67_ | 8 | 0.605 | 0.574, 0.636 | <10^-8^ |  |  |  |  |  |  |  |  |  |  | 0.046 | -21.3 | 5.3 |
| L_T_ | 8 | -2.281 | -3.959, -0.603 | 0.045 | 0.846 | 0.760, 0.932 | <10^-5^ |  |  |  | 95.621 | 35.745, 155.477 | 0.026 |  | 0.025 | -18.9 | 7.7 |
| C_T_ | 8 | -1.980 | -5.287, 1.327 | 0.306 | 0.613 | -1.508, 2.734 | 0.601 | 0.059 | -0.474, 0.592 | 0.839 | 92.850 | -21.718, 163.982 | 0.063 |  | 0.026 | -0.3 | *n.s.* |
| L_0.75,T_ | 8 | -1.403 | -3.298, 0.492 | 0.197 |  |  |  |  |  |  | 69.350 | -1.043, 139.743 | 0.102 |  | 0.032 | -22.8 | *n.s.* |
| L_0.67,T_ | 8 | -0.675 | -3.452, 2.102 | 0.651 |  |  |  |  |  |  | 47.567 | -55.652, 150.786 | 0.401 |  | 0.046 | -16.7 | *n.s.* |
| L_PGLS_ | 8 | 0.293 | 0.230, 0.356 | <10^-4^ | 0.849 | 0.849, 0.849 | <10^-16^ |  |  |  |  |  |  | 1.705 | 0.061 | -26.6 | **0** |
| C_PGLS_ | 8 | 1.407 | 0.421, 2.393 | 0.038 | -0.242 | -1.459, 0.975 | 0.713 | 0.260 | -0.103, 0.623 | 0.219 |  |  |  | 1.705 | 0.062 | -7.5 | *n.s.* |
| L_0.75,PGLS_ | 8 | 0.471 | 0.438, 0.504 | <10^-7^ |  |  |  |  |  |  |  |  |  | 0.623 | 0.039 | -19.0 | 7.6 |
| L_0.67,PGLS_ | 8 | 0.619 | 0.572, 0.666 | <10^-7^ |  |  |  |  |  |  |  |  |  | 1.069 | 0.049 | -16.1 | 10.5 |
| L_T,PGLS_ | 8 | -7.080 | -9.052, -5.108 | 0.001 | 0.940 | 0.607, 1.273 | 0.003 |  |  |  | 267.919 | 198.656, 337.181 | <10^-3^ | 2.006 | 0.103 | 17.8 | 8.8 |
| C_T,PGLS_ | 8 | -1.640 | -4.600, 1.320 | 0.339 | 1.882 | -0.323, 4.087 | 0.170 | -0.269 | -0.839, 0.301 | 0.407 | 35.902 | -2.203, 69.600 | 0.105 | 1.705 | 0.048 | 33.2 | *n.s.* |
| L_0.75,T,PGLS_ | 8 | -0.066 | -0.123, 0.051 | 0.066 |  |  |  |  |  |  | 20.079 | 20.078, 20.080 | <10^-16^ | 1.705 | 0.057 | -31.1 | *n.s.* |
| L_0.67,T,PGLS_ | 8 | -0.356 | -0.421, -0.291 | <10^-4^ |  |  |  |  |  |  | 36.270 | 36.262, 36.278 | <10^-16^ | 1.705 | 0.065 | -26.1 | 0.5 |

Table S11. Metabolic scaling in Afrosoricida. Data set on Afrosoricida is a subset from Sieg *et al.* (2009). It comprises those species for which temperature and phylogenetic information is available. Estimated beta values (ß.), their 95% confidence intervals (95%CI), their p-values, residual standard errors of models (Res.Se.), AICc and ΔAIC values of models are listed for all 16 models studied. ß_0_ = intercept, ß_1_ = slope, ß_2_ = coefficient of the quadratic term, ß_3_ = coefficient of the temperature term, λ = strength of the phylogenetic signal (only PGLS). *n.s*.: non-significant model, *div*: no convergence of fitting, *inf.*: infinity.

| model | N | *ß_0_* | 95%CI | p | *ß_1_* | 95%CI | p | *ß_2_* | 95%CI | p | *ß_3_* | 95%CI | p | λ | Res.S.E. | AICc | ΔAIC |
| --- | --- | --- | --- | --- | --- | --- | --- | --- | --- | --- | --- | --- | --- | --- | --- | --- | --- |
| L | 9 | 0.577 | 0.209, 0.945 | 0.015 | 0.625 | 0.425, 0.824 | <10^-3^ |  |  |  |  |  |  |  | 0.199 | 3.8 | 4.0 |
| C | 9 | 0.064 | -0.953, 1.091 | 0.906 | 1.235 | -0.077, 2.393 | 0.075 | -0.161 | -0.461, 0.139 | 0.329 |  |  |  |  | 0.197 | 8.4 | *n.s.* |
| L_0.75_ | 9 | 0.360 | 0.233, 0.487 | <10^-3^ |  |  |  |  |  |  |  |  |  |  | 0.204 | 1.3 | 1.5 |
| L_0.67_ | 9 | 0.499 | 0.381, 0.617 | <10^-4^ |  |  |  |  |  |  |  |  |  |  | 0.190 | -0.2 | **0** |
| L_T_ | 9 | 1.843 | -1.830, 5.516 | 0.358 | 0.612 | 0.402, 0.822 | 0.001 |  |  |  | -40.290 | -99.595, 75.948 | 0.519 |  | 0.206 | 9.2 | *n.s.* |
| C_T_ | 9 | 1.127 | -2.901, 5.155 | 0.603 | 1.178 | -0.061, 2.417 | 0.112 | -0.149 | -0.470, 0.172 | 0.399 | -32.560 | -151.456, 86.336 | 0.611 |  | 0.208 | 16.9 | *n.s.* |
| L_0.75,T_ | 9 | 1.192 | -2.485, 4.869 | 0.543 | 1.570 | -1.805, 4.945 | 0.389 |  |  |  | -26.945 | -145.942, 92.052 | 0.669 |  | 0.214 | 5.3 | *n.s.* |
| L_0.67,T_ | 9 | 1.570 | -1.805, 4.945 | 0.389 |  |  |  |  |  |  | -34.689 | -143.937, 74.559 | 0.551 |  | 0.197 | 3.6 | *n.s.* |
| L_PGLS_ | 9 |  |  |  |  |  |  |  |  |  |  |  |  |  |  |  | *div.* |
| C_PGLS_ | 9 |  |  |  |  |  |  |  |  |  |  |  |  |  | 0.298 |  | *div.* |
| L_0.75,PGLS_ | 9 | 0.395 | 0.215, 0.575 | 0.003 |  |  |  |  |  |  |  |  |  | 0.512 | 0.199 | 5.3 | 5.5 |
| L_0.67,PGLS_ | 9 | 0.536 | 0.397, 0.675 | <10^-4^ |  |  |  |  |  |  |  |  |  | 0.314 | 0.171 | 3.1 | 3.3 |
| L_T,PGLS_ | 9 |  |  |  |  |  |  |  |  |  |  |  |  |  |  |  | *div.* |
| C_T,PGLS_ | 9 |  |  |  |  |  |  |  |  |  |  |  |  |  |  |  | *div.* |
| L_0.75,T,PGLS_ | 9 | 4.363 | 3.585, 5.141 | <10^-5^ |  |  |  |  |  |  | -129.012 | -152.595, -105.429 | <10^-5^ | 1.209 | 0.252 | 5.0 | 5.2 |
| L_0.67,T,PGLS_ | 9 | 3.795 | 3.080, 4.510 | <10^-4^ |  |  |  |  |  |  | -105.920 | -127.758, -84.082 | <10^-4^ | 1.208 | 0.219 | 2.7 | 2.9 |

Table S12. Metabolic scaling in Rodentia. Data set on Rodentia is a subset from Sieg *et al.* (2009). It comprises those species for which temperature and phylogenetic information is available. Estimated beta values (ß.), their 95% confidence intervals (95%CI), their p-values, residual standard errors of models (Res.Se.), AICc and ΔAIC values of models are listed for all 16 models studied. ß_0_ = intercept, ß_1_ = slope, ß_2_ = coefficient of the quadratic term, ß_3_ = coefficient of the temperature term, λ = strength of the phylogenetic signal (only PGLS). *n.s*.: non-significant model, *div*: no convergence of fitting, *inf.*: infinity.

| model | N | *ß_0_* | 95%CI | p | *ß_1_* | 95%CI | p | *ß_2_* | 95%CI | p | *ß_3_* | 95%CI | p | λ | Res.S.E. | AICc | ΔAIC |
| --- | --- | --- | --- | --- | --- | --- | --- | --- | --- | --- | --- | --- | --- | --- | --- | --- | --- |
| L | 236 | 0.678 | 0.617, 0.739 | <10^-16^ | 0.673 | 0.644, 0.702 | <10^-16^ |  |  |  |  |  |  |  | 0.149 | -227.8 | 44.4 |
| C | 236 | 0.835 | 0.670, 1.000 | <10^-16^ | 0.523 | 0.372, 0.674 | <10^-10^ | 0.033 | 0.001, 0.064 | 0.047 |  |  |  |  | 0.148 | -229.8 | 42.4 |
| L_0.75_ | 236 | 0.527 | 0.507, 0.547 | <10^-16^ |  |  |  |  |  |  |  |  |  |  | 0.157 | -205.3 | 66.9 |
| L_0.67_ | 236 | 0.685 | 0.665, 0.705 | <10^-16^ |  |  |  |  |  |  |  |  |  |  | 0.149 | -229.8 | 42.4 |
| L_T_ | 236 | 1.732 | 1.230, 2.234 | <10^-10^ | 0.670 | 0.641, 0.699 | <10^-16^ |  |  |  | -38.493 | -56.721, -20.265 | <10^-4^ |  | 0.144 | -242.5 | 29.7 |
| C_T_ | 236 | 1.875 | 1.356, 2.394 | <10^-11^ | 0.525 | 0.380, 0.670 | <10^-10^ | 0.032 | 0.001, 0.063 | 0.047 | -38.186 | -56.302, -20.070 | <10^-4^ |  | 0.144 | -244.5 | 27.7 |
| L_0.75,T_ | 236 | 1.502 | 0.977, 2.027 | <10^-7^ |  |  |  |  |  |  | -35.804 | -55.071, -16,573 | <10^-3^ |  | 0.153 | -216.3 | 55.9 |
| L_0.67,T_ | 236 | 1.732 | 1.238, 2.226 | <10^-10^ |  |  |  |  |  |  | -38.494 | -56.657, -20.331 | <10^-4^ |  | 0.144 | -244.6 | 27.6 |
| L_PGLS_ | 236 | 0.608 | 0.494, 0.722 | <10^-16^ | 0.700 | 0.663, 0.737 | <10^-16^ |  |  |  |  |  |  | 0.521 | 0.161 | -271.0 | 1.2 |
| C_PGLS_ | 236 | 0.858 | 0.691, 1.025 | <10^-16^ | 0.511 | 0.362, 0.660 | <10^-10^ | 0.036 | 0.005, 0.067 | 0.025 |  |  |  | 0.094 | 0.147 | -235.7 | 36.5 |
| L_0.75,PGLS_ | 236 | 0.501 | 0.417, 0.585 | <10^-16^ |  |  |  |  |  |  |  |  |  | 0.561 | 0.167 | -266.6 | 5.6 |
| L_0.67,PGLS_ | 236 | 0.674 | 0.596, 0.752 | <10^-16^ |  |  |  |  |  |  |  |  |  | 0.515 | 0.161 | -270.6 | 2.2 |
| L_T,PGLS_ | 236 | 1.101 | 0.558, 1.644 | <10^-4^ | 0.696 | 0.659, 0.733 | <10^-16^ |  |  |  | -17.694 | -36.847, 1.459 | 0.071 | 0.471 | 0.156 | -272.0 | *n.s.* |
| C_T,PGLS_ | 236 | 1.833 | 1.304, 2.362 | <10^-10^ | 0.511 | 0.366, 0.656 | <10^-10^ | 0.035 | 0.004, 0.066 | 0.025 | -35.859 | -54.381, -17.337 | <10^-3^ | 0.049 | 0.142 | -247.2 | 25.2 |
| L_0.75,T,PGLS_ | 236 | 0.873 | 0.338, 1.408 | 0.002 |  |  |  |  |  |  | -13.610 | -32.941, 5.721 | 0.169 | 0.533 | 0.164 | -266.4 | *n.s.* |
| L_0.67,T,PGLS_ | 236 | 1.204 | 0.679, 1.729 | <10^-5^ |  |  |  |  |  |  | -19.398 | -38.465, -0.331 | 0.047 | 0.458 | 0.156 | -272.2 | **0** |

Table S13. Metabolic scaling in Erinaceomorpha. Data set on Erinaceomorpha is a subset from Sieg *et al.* (2009). It comprises those species for which temperature and phylogenetic information is available. Estimated beta values (ß.), their 95% confidence intervals (95%CI), their p-values, residual standard errors of models (Res.Se.), AICc and ΔAIC values of models are listed for all 16 models studied. ß_0_ = intercept, ß_1_ = slope, ß_2_ = coefficient of the quadratic term, ß_3_ = coefficient of the temperature term, λ = strength of the phylogenetic signal (only PGLS). *n.s*.: non-significant model, *div*: no convergence of fitting, *inf.*: infinity.

| model | N | *ß_0_* | 95%CI | p | *ß_1_* | 95%CI | p | *ß_2_* | 95%CI | p | *ß_3_* | 95%CI | p | λ | Res.S.E. | AICc | ΔAIC |
| --- | --- | --- | --- | --- | --- | --- | --- | --- | --- | --- | --- | --- | --- | --- | --- | --- | --- |
| L | 7 | 0.529 | -0.574, 1.632 | 0.391 | 0.669 | 0.255, 1.083 | 0.025 |  |  |  |  |  |  |  | 0.188 | 8.1 | 14.1 |
| C | 7 | 6.430 | -0.611, 12.249 | 0.096 | -4.369 | -9.291, 0.553 | 0.157 | 1.044 | -0.027, 2.061 | 0.115 |  |  |  |  | 0.148 | 17.2 | *n.s.* |
| L_0.75_ | 7 | 0.314 | 0.185, 0.443 | 0.003 |  |  |  |  |  |  |  |  |  |  | 0.174 | 1.3 | 7.3 |
| L_0.67_ | 7 | 0.526 | 0.399, 0.653 | <10^-3^ |  |  |  |  |  |  |  |  |  |  | 0.172 | 1.1 | 7.1 |
| L_T_ | 7 | 4.440 | 1.356, 7.523 | 0.048 | 0.934 | 0.585, 1.283 | 0.006 |  |  |  | -161.909 | -285.632, 38.186 | 0.062 |  | 0.129 | 15.3 | *n.s.* |
| C_T_ | 7 | 6.837 | -2.037, 11.637 | 0.068 | -2.080 | -6.896, 2.736 | 0.460 | 0.619 | -0.353, 1.591 | 0.307 | -118.443 | -254.002, 17.116 | 0.185 |  | 0.122 | 54.5 | *n.s.* |
| L_0.75,T_ | 7 | 3.849 | 0.958, 6.740 | 0.048 |  |  |  |  |  |  | -124.057 | -225.499, 22.615 | 0.062 |  | 0.130 | 3.0 | *n.s.* |
| L_0.67,T_ | 7 | 3.591 | -0.392, 6.790 | 0.079 |  |  |  |  |  |  | -107.586 | -219.782, 4.610 | 0.119 |  | 0.144 | 4.4 | *n.s.* |
| L_PGLS_ | 7 | -0.016 | -1.129, 1.097 | 0.979 | 0.909 | 0.482, 1.336 | 0.009 |  |  |  |  |  |  | 1.140 | 0.257 | 18.7 | 24.7 |
| C_PGLS_ | 7 |  |  |  |  |  |  |  |  |  |  |  |  |  |  |  | *div.* |
| L_0.75,PGLS_ | 7 | 0.384 | 0.076, 0.692 | 0.050 |  |  |  |  |  |  |  |  |  | 1.149 | 0.260 | 5.2 | 11.2 |
| L_0.67,PGLS_ | 7 | 0.584 | 0.251, 0.917 | 0.014 |  |  |  |  |  |  |  |  |  | 1.152 | 0.281 | 5.7 | 11.7 |
| L_T,PGLS_ | 7 | 0.844 | 0.313, 1.375 | 0.036 | 1.018 | 0.555, 1.481 | 0.013 |  |  |  | -40.452 | -66.455, -14.449 | 0.038 | 1.156 | 0.260 | 35.6 | 41.6 |
| C_T,PGLS_ | 7 | 7.971 | 5.100, 10.842 | 0.012 | -2.342 | -5.982, 1.298 | 0.296 | 0.686 | -0.047, 1.419 | 0.164 | -153.079 | -282,392, 23.766 | 0.103 | -1.149 | 0.064 | *+inf* | *n.s.* |
| L_0.75,T,PGLS_ | 7 | 1.094 | 0.776, 1.412 | 0.001 |  |  |  |  |  |  | -25.394 | -25.394, -25.394 | <10^-16^ | 1.156 | 0.267 | -6.0 | **0** |
| L_0.67,T,PGLS_ | 7 | 1.169 | 0.826, 1.512 | 0.001 |  |  |  |  |  |  | -20.898 | -20.898, -20.898 | <10^-16^ | 1.156 | 0.289 | -4.4 | 1.6 |

Table S14. Metabolic scaling in Primates. Data set on Primates is a subset from Sieg *et al.* (2009). It comprises those species for which temperature and phylogenetic information is available. Estimated beta values (ß.), their 95% confidence intervals (95%CI), their p-values, residual standard errors of models (Res.Se.), AICc and ΔAIC values of models are listed for all 16 models studied. ß_0_ = intercept, ß_1_ = slope, ß_2_ = coefficient of the quadratic term, ß_3_ = coefficient of the temperature term, λ = strength of the phylogenetic signal (only PGLS). *n.s*.: non-significant model, *div*: no convergence of fitting, *inf.*: infinity.

| model | N | *ß_0_* | 95%CI | p | *ß_1_* | 95%CI | p | *ß_2_* | 95%CI | p | *ß_3_* | 95%CI | p | Λ | Res.S.E. | AICc | ΔAIC |
| --- | --- | --- | --- | --- | --- | --- | --- | --- | --- | --- | --- | --- | --- | --- | --- | --- | --- |
| L | 18 | 0.513 | 0.139, 0.887 | 0.015 | 0.721 | 0.598, 0.844 | <10^-9^ |  |  |  |  |  |  |  | 0.188 | -4.1 | 2.6 |
| C | 18 | 2,101 | 0.692, 3.510 | 0.001 | -0.377 | -1.851, 0.576 | 0.449 | 0.180 | -0.025, 0.335 | 0.073 |  |  |  |  | 0.168 | -6.2 | *n.s.* |
| L_0.75_ | 18 | 0.427 | 0.345, 0.509 | <10^-8^ |  |  |  |  |  |  |  |  |  |  | 0.184 | -6.7 | **0** |
| L_0.67_ | 18 | 0.665 | 0.581, 0.749 | <10^-11^ |  |  |  |  |  |  |  |  |  |  | 0.186 | -6.2 | 0.5 |
| L_T_ | 18 | 1.884 | -0.609, 4.377 | 0.158 | 0.705 | 0.580, 0.830 | <10^-8^ |  |  |  | -48.968 | -137.017, 39.081 | 0.292 |  | 0.187 | -2.2 | *n.s.* |
| C_T_ | 18 | 4.309 | 1.590, 7.030 | 0.007 | -0.568 | -1.481, 0.345 | 0.242 | 0.207 | 0.060, 0.354 | 0.015 | -70.200 | -145.885, 5.485 | 0.090 |  | 0.158 | -6.2 | *n.s.* |
| L_0.75,T_ | 18 | 1.553 | -0.729, 3.833 | 0.200 |  |  |  |  |  |  | -41.641 | -126.017, 42.735 | 0.347 |  | 0.184 | -4.9 | *n.s.* |
| L_0.67,T_ | 18 | 2.142 | -0.126, 4.410 | 0.082 |  |  |  |  |  |  | -54.664 | -138.538, 29.210 | 0.219 |  | 0.183 | -5.1 | *n.s.* |
| L_PGLS_ | 18 | 0.588 | 0.163, 1.013 | 0.003 | 0.686 | 0.543, 0.829 | <10^-7^ |  |  |  |  |  |  | 0.202 | 0.194 | -0.4 | 5.8 |
| C_PGLS_ | 18 |  |  |  |  |  |  |  |  |  |  |  |  |  |  |  | *div.* |
| L_0.75,PGLS_ | 18 | 0.422 | 0.334, 0.510 | <10^-7^ |  |  |  |  |  |  |  |  |  | 0.011 | 0.187 | -2.3 | 4.4 |
| L_0.67,PGLS_ | 18 | 0.632 | 0.514, 0.748 | <10^-8^ |  |  |  |  |  |  |  |  |  | 0.243 | 0.190 | -2.9 | 3.8 |
| L_T,PGLS_ | 18 |  |  |  |  |  |  |  |  |  |  |  |  |  |  |  | *div.* |
| C_T,PGLS_ | 18 |  |  |  |  |  |  |  |  |  |  |  |  |  |  |  | *div.* |
| L_0.75,T,PGLS_ | 18 |  |  |  |  |  |  |  |  |  |  |  |  |  |  |  | *div.* |
| L_0.67,T,PGLS_ | 18 |  |  |  |  |  |  |  |  |  |  |  |  |  |  |  | *div.* |

Table S15. Metabolic scaling in Lagomorpha. Data set on Lagomorpha is a subset from Sieg *et al.* (2009). It comprises those species for which temperature and phylogenetic information is available. Estimated beta values (ß.), their 95% confidence intervals (95%CI), their p-values, residual standard errors of models (Res.Se.), AICc and ΔAIC values of models are listed for all 16 models studied. ß_0_ = intercept, ß_1_ = slope, ß_2_ = coefficient of the quadratic term, ß_3_ = coefficient of the temperature term, λ = strength of the phylogenetic signal (only PGLS). *n.s*.: non-significant model, *div*: no convergence of fitting, *inf.*: infinity.

| model | N | *ß_0_* | 95%CI | p | *ß_1_* | 95%CI | p | *ß_2_* | 95%CI | p | *ß_3_* | 95%CI | p | λ | Res.S.E. | AICc | ΔAIC |
| --- | --- | --- | --- | --- | --- | --- | --- | --- | --- | --- | --- | --- | --- | --- | --- | --- | --- |
| L | 11 | 0.813 | 0.495, 1.131 | <10^-3^ | 0.690 | 0.586, 0.794 | <10^-6^ |  |  |  |  |  |  |  | 0.092 | -14.1 | 13.0 |
| C | 11 | 0.898 | -1.403, 3.199 | 0.466 | 0.627 | -1.072, 2.326 | 0.490 | 0.011 | -0.291, 0.313 | 0.944 |  |  |  |  | 0.098 | -8.9 | *n.s.* |
| L_0.75_ | 11 | 0.632 | 0.577, 0.687 | <10^-9^ |  |  |  |  |  |  |  |  |  |  | 0.093 | -16.6 | 10.5 |
| L_0.67_ | 11 | 0.875 | 0.824, 0.926 | <10^-10^ |  |  |  |  |  |  |  |  |  |  | 0.088 | -17.9 | 9.2 |
| L_T_ | 11 | 2.545 | -0.174, 5.264 | 0.104 | 0.690 | 0.590, 0.790 | <10^-6^ |  |  |  | -67.241 | -172.121, 37.689 | 0.244 |  | 0.089 | -10.9 | *n.s.* |
| C_T_ | 11 | 2.543 | -1.009, 6.095 | 0.203 | 0.692 | -0.974, 2.358 | 0.442 | -0.0003 | -0.296, 0.296 | 0.998 | -67.254 | -179.615, 45.120 | 0.279 |  | 0.095 | -3.5 | *n.s.* |
| L_0.75,T_ | 11 | 2.357 | -0.399, 5.113 | 0.128 |  |  |  |  |  |  | -67.018 | -174.095, 40.059 | 0.251 |  | 0.091 | -14.4 | *n.s.* |
| L_0.67,T_ | 11 | 2.608 | -0.038, 5.178 | 0.078 |  |  |  |  |  |  | -67.315 | -167.157, 32.527 | 0.219 |  | 0.085 | -15.9 | *n.s.* |
| L_PGLS_ | 11 |  |  |  |  |  |  |  |  |  |  |  |  |  |  |  | *div.* |
| C_PGLS_ | 11 | 1.228 | 0.270, 2.186 | 0.036 | 0.471 | -0.433, 1.375 | 0.336 | 0.018 | -0.172, 0.208 | 0.856 |  |  |  | 1.103 | 0.120 | -16.9 | *n.s.* |
| L_0.75,PGLS_ | 11 | 0.621 | 0.621, 0.621 | <10^-16^ |  |  |  |  |  |  |  |  |  | -0.183 | 0.088 | -27.1 | **0** |
| L_0.67,PGLS_ | 11 |  |  |  |  |  |  |  |  |  |  |  |  |  |  |  | *div.* |
| L_T,PGLS_ | 11 |  |  |  |  |  |  |  |  |  |  |  |  |  |  |  | *div.* |
| C_T,PGLS_ | 11 | 1.441 | -0.572, 3.454 | 0.203 | -0.191 | -2.208, 1.826 | 0.858 | 0.133 | -0.210, 0.476 | 0.473 | 24.672 | -6.958, 56.302 | 0.170 | 1.628 | 0.144 | -0.462 | *n.s.* |
| L_0.75,T,PGLS_ | 11 |  |  |  |  |  |  |  |  |  |  |  |  |  |  |  | *div.* |
| L_0.67,T,PGLS_ | 11 |  |  |  |  |  |  |  |  |  |  |  |  |  |  |  | *div.* |

Table S16. Metabolic scaling in Pilosa. Data set on Pilosa is a subset from Sieg *et al.* (2009). It comprises those species for which temperature and phylogenetic information is available. Estimated beta values (ß.), their 95% confidence intervals (95%CI), their p-values, residual standard errors of models (Res.Se.), AICc and ΔAIC values of models are listed for all 16 models studied. ß_0_ = intercept, ß_1_ = slope, ß_2_ = coefficient of the quadratic term, ß_3_ = coefficient of the temperature term, λ = strength of the phylogenetic signal (only PGLS). *n.s*.: non-significant model, *div*: no convergence of fitting, *inf.*: infinity.

| model | N | *ß_0_* | 95%CI | p | *ß_1_* | 95%CI | p | *ß_2_* | 95%CI | p | *ß_3_* | 95%CI | p | λ | Res.S.E. | AICc | ΔAIC |
| --- | --- | --- | --- | --- | --- | --- | --- | --- | --- | --- | --- | --- | --- | --- | --- | --- | --- |
| L | 6 | 0.590 | 0.237, 0.943 | 0.031 | 0.641 | 0.543, 0.739 | <10^-3^ |  |  |  |  |  |  |  | 0.077 | 1.8 | 9.2 |
| C | 6 | -0.166 | -1.438, 1.106 | 0.815 | 1.106 | -0.346, 1.866 | 0.065 | -0.069 | -0.181, 0.043 | 0.314 |  |  |  |  | 0.073 | 29.4 | *n.s.* |
| L_0.75_ | 6 | 0.207 | 0.127, 0.287 | 0.004 |  |  |  |  |  |  |  |  |  |  | 0.101 | -3.6 | 7.4 |
| L_0.67_ | 6 | 0.488 | 0.431, 0.549 | <10^-5^ |  |  |  |  |  |  |  |  |  |  | 0.071 | -7.7 | 3.3 |
| L_T_ | 6 | 1.137 | -1.930, 4.204 | 0.520 | 0.645 | 0.531, 0.759 | 0.002 |  |  |  | -18.468 | -121.203, 84.267 | 0.748 |  | 0.087 | 31.5 | *n.s.* |
| C_T_ | 6 | -0.095 | -4.182, 3.992 | 0.968 | 1.100 | -0.076, 2.276 | 0.157 | -0.068 | -0.213, 0.302 | 0.453 | -2.083 | -112.975, 108.809 | 0.974 |  | 0.089 | *+inf* | *n.s.* |
| L_0.75,T_ | 6 | 1.258 | -2.591, 5.107 | 0.557 |  |  |  |  |  |  | -34.726 | -161.905, 92.453 | 0.621 |  | 0.109 | 6.0 | *n.s.* |
| L_0.67,T_ | 6 | 1.166 | -1.572, 3.904 | 0.451 |  |  |  |  |  |  | -22.388 | -122.828, 68.052 | 0.653 |  | 0.077 | 1.9 | *n.s.* |
| L_PGLS_ | 6 | 0.536 | 0.371, 0.701 | 0.003 | 0.651 | 0.606, 0.696 | <10^-5^ |  |  |  |  |  |  | -0.940 | 0.032 | 19.4 | 8.4 |
| C_PGLS_ | 6 | -0.098 | -0.512, 0.316 | 0.674 | 1.063 | 0.824, 1.302 | 0.003 | -0.062 | -0.097, -0.027 | 0.040 |  |  |  | 1.261 | 0.075 | *+inf* | *+inf* |
| L_0.75,PGLS_ | 6 | 0.192 | 0.186, 0.198 | <10^-8^ |  |  |  |  |  |  |  |  |  | -2.879 | 0.058 | -0.3 | 10.7 |
| L_0.67,PGLS_ | 6 | 0.466 | 0.464, 0.470 | <10^-12^ |  |  |  |  |  |  |  |  |  | -0.974 | 0.028 | -11.0 | **0** |
| L_T,PGLS_ | 6 | 3.048 | 2.877, 3.219 | <10^-4^ | 0.548 | 0.475, 0.621 | <10^-3^ |  |  |  | -71.865 | -79.703, -64.027 | <10^-3^ | 1.261 | 0.141 | *+inf* | *+inf* |
| C_T,PGLS_ | 6 | 1.315 | -4.073, 6.703 | 0.680 | 0.761 | -1.793, 3.315 | 0.618 | -0.026 | -0.342, 0.290 | 0.886 | -26.211 | -99.035, 46.613 | 0.554 | 1.306 | 0.082 | -102.6 | *n.s.* |
| L_0.75,T,PGLS_ | 6 |  |  |  |  |  |  |  |  |  |  |  |  |  |  |  | *div.* |
| L_0.67,T,PGLS_ | 6 | 0.391 | -0.460, 1.242 | 0.419 |  |  |  |  |  |  | 2.521 | -25.131, 30.173 | 0.867 | -0.940 | 0.035 | 20.3 | *n.s.* |

Table S17. Metabolic scaling in Cingulata. Data set on Cingulata is a subset from Sieg *et al.* (2009). It comprises those species for which temperature and phylogenetic information is available. Estimated beta values (ß.), their 95% confidence intervals (95%CI), their p-values, residual standard errors of models (Res.Se.), AICc and ΔAIC values of models are listed for all 16 models studied. ß_0_ = intercept, ß_1_ = slope, ß_2_ = coefficient of the quadratic term, ß_3_ = coefficient of the temperature term, λ = strength of the phylogenetic signal (only PGLS). *n.s*.: non-significant model, *div*: no convergence of fitting, *inf.*: infinity.

| model | N | *ß_0_* | 95%CI | p | *ß_1_* | 95%CI | p | *ß_2_* | 95%CI | p | *ß_3_* | 95%CI | p | λ | Res.S.E. | AICc | ΔAIC |
| --- | --- | --- | --- | --- | --- | --- | --- | --- | --- | --- | --- | --- | --- | --- | --- | --- | --- |
| L | 9 | 0.452 | -0.028, 0.932 | 0.108 | 0.669 | 0.536, 0.802 | <10^-4^ |  |  |  |  |  |  |  | 0.096 | -8.0 | 17.8 |
| C | 9 | -2.948 | -5.610, 0.286 | 0.073 | 2.483 | 1.072, 3.894 | 0.014 | -0.237 | -0.421, -0.053 | 0.045 |  |  |  |  | 0.073 | -7.3 | 18.5 |
| L_0.75_ | 9 | 0.163 | 0.098, 0.228 | 0.001 |  |  |  |  |  |  |  |  |  |  | 0.099 | -11.2 | 14.6 |
| L_0.67_ | 9 | 0.448 | 0.389, 0.507 | <10^-6^ |  |  |  |  |  |  |  |  |  |  | 0.090 | -12.8 | 13.0 |
| L_T_ | 9 | 2.561 | -0.158, 4.964 | 0.082 | 0.691 | 0.571, 0.811 | <10^-4^ |  |  |  | -75.059 | -159.284, 9.166 | 0.133 |  | 0.085 | -4.5 | *n.s.* |
| C_T_ | 9 | -1.013 | -5.158, 3.132 | 0.652 | 2.145 | 0.663, 3.627 | 0.036 | -0.191 | -0.385, 0.004 | 0.112 | -45.455 | -121.546, 30.636 | 0.294 |  | 0.070 | 2.5 | *n.s.* |
| L_0.75,T_ | 9 | 2.597 | -0.206, 4.988 | 0.071 |  |  |  |  |  |  | -83.550 | -165.634, 1.465 | 0.086 |  | 0.084 | -10.4 | *n.s.* |
| L_0.67,T_ | 9 | 2.548 | -0.302, 4.794 | 0.061 |  |  |  |  |  |  | -72.100 | -149.155, 4.955 | 0.109 |  | 0.079 | -11.6 | *n.s.* |
| L_PGLS_ | 9 | 0.612 | 0.569, 0.655 | <10^-7^ | 0.658 | 0.634, 0.682 | <10^-9^ |  |  |  |  |  |  | 4.690 | 0.036 | -19.9 | 5.9 |
| C_PGLS_ | 9 | -3.730 | -5.804, -1.656 | 0.012 | 2.891 | 1.805, 3.977 | 0.002 | -0.291 | -0.432, -0.150 | 0.007 |  |  |  | 0.838 | 0.077 | 2.2 | 23.6 |
| L_0.75,PGLS_ | 9 | 0.177 | -0.012, 0.342 | 0.069 |  |  |  |  |  |  |  |  |  | 1.146 | 0.146 | -8.8 | *n.s.* |
| L_0.67,PGLS_ | 9 | 0.585 | 0.583, 0.587 | <10^-16^ |  |  |  |  |  |  |  |  |  | 4.514 | 0.036 | -25.8 | **0** |
| L_T,PGLS_ | 9 | 0.625 | 0.603, 0.647 | <10^-8^ | 0.662 | 0.628, 0.699 | <10^-7^ |  |  |  | -1.001 | -5.337, 3.335 | 0.667 | 4.984 | 0.036 | -9.9 | *n.s.* |
| C_T,PGLS_ | 9 | -3.810 | -8.461, 0.841 | 0.169 | 2.908 | 1.456, 4.360 | 0.011 | -0.294 | -0.486, -0.102 | 0.030 | 1.679 | -85.429, 88.787 | 0.971 | 0.852 | 0.085 | 26.2 | *n.s.* |
| L_0.75,T,PGLS_ | 9 | 1.682 | -0.664, 4.028 | 0.203 |  |  |  |  |  |  | -51.380 | -131.326, 28.566 | 0.248 | 1.128 | 0.136 | -3.4 | *n.s.* |
| L_0.67,T,PGLS_ | 9 | 1.801 | -0.573, 4.175 | 0.180 |  |  |  |  |  |  | -45.582 | -126.452, 35.388 | 0.306 | 1.089 | 0.122 | -4.2 | *n.s.* |

Table S18. Metabolic scaling in Pholidota. Data set on Pholidota is a subset from Sieg *et al.* (2009). It comprises those species for which temperature and phylogenetic information is available. Estimated beta values (ß.), their 95% confidence intervals (95%CI), their p-values, residual standard errors of models (Res.Se.), AICc and ΔAIC values of models are listed for all 16 models studied. ß_0_ = intercept, ß_1_ = slope, ß_2_ = coefficient of the quadratic term, ß_3_ = coefficient of the temperature term, λ = strength of the phylogenetic signal (only PGLS). *n.s*.: non-significant model, *div*: no convergence of fitting, *inf.*: infinity.

| model | N | *ß_0_* | 95%CI | p | *ß_1_* | 95%CI | p | *ß_2_* | 95%CI | p | *ß_3_* | 95%CI | p | λ | Res.S.E. | AICc | ΔAIC |
| --- | --- | --- | --- | --- | --- | --- | --- | --- | --- | --- | --- | --- | --- | --- | --- | --- | --- |
| L | 5 | 1.113 | -0.198, 2.028 | 0.097 | 0.487 | 0.230, 0.744 | 0.034 |  |  |  |  |  |  |  | 0.112 | 19.8 | 122.6 |
| C | 5 | -5.227 | -14.190, 3.736 | 0.371 | 3.991 | -0.948, 8.930 | 0.254 | -0.478 | -1.152, 0.196 | 0.299 |  |  |  |  | 0.098 | +inf | *n.s.* |
| L_0.75_ | 5 | 0.181 | -0.050, 0.312 | 0.053 |  |  |  |  |  |  |  |  |  |  | 0.149 | 4.0 | *n.s.* |
| L_0.67_ | 5 | 0.465 | 0.355, 0.575 | 0.001 |  |  |  |  |  |  |  |  |  |  | 0.125 | 2.3 | 105.1 |
| L_T_ | 5 | -5.603 | -10.615, 0.591 | 0.160 | 0.582 | 0.417, 0.747 | 0.020 |  |  |  | 210.135 | -54.229, 366.544 | 0.118 |  | 0.065 | +inf | *n.s.* |
| C_T_ | 5 | -8.990 | -9.280, -8.700 | 0.011 | 3.023 | 2.880, 3.166 | 0.015 | -0.335 | -0.355, -0.315 | 0.019 | 177.015 | 170.131, 183.899 | 0.013 |  | 0.003 | -102.8 | **0** |
| L_0.75,T_ | 5 | -8.272 | -14.328, 2.216 | 0.075 |  |  |  |  |  |  | 278.403 | -78.928, 477.878 | 0.072 |  | 0.092 | 17.8 | *n.s.* |
| L_0.67,T_ | 5 | -7.002 | -11.353, 2.651 | 0.051 |  |  |  |  |  |  | 245.914 | 102.634, 389.194 | 0.044 |  | 0.066 | 14.5 | *n.s.* |
| L_PGLS_ | 5 | 1.349 | 1.190, 1.508 | <10^-3^ | 0.420 | 0.420, 0.420 | <10^-16^ |  |  |  |  |  |  | 1.785 | 0.139 | *+inf* | *+inf* |
| C_PGLS_ | 5 | -10.697 | -15.136, -6.258 | 0.042 | 7.021 | 4.704, 9.338 | 0.027 | -0.890 | -1.186, -0.594 | 0.028 |  |  |  | 3.164 | 0.058 | -71.4 | 31.4 |
| L_0.75,PGLS_ | 5 | 0.125 | 0.125, 0.125 | <10^-16^ |  |  |  |  |  |  |  |  |  | -1.261 | 0.148 | -4.6 | 98.2 |
| L_0.67,PGLS_ | 5 | 0.447 | 0.447, 0.447 | <10^-16^ |  |  |  |  |  |  |  |  |  | -3.293 | 0.096 | 7.1 | 109.9 |
| L_T,PGLS_ | 5 | -7.615 | -10.241, -4.989 | 0.030 | 0.558 | 0.448, 0.668 | 0.010 |  |  |  | 278.174 | 204.400, 351.948 | 0.018 | 3.099 | 0.039 | -75.0 | 177.8 |
| C_T,PGLS_ | 5 | -8.937 | -9.221, -8.653 | 0.010 | 2.988 | 2.694, 3.282 | 0.032 | -0.330 | -0.371, -0.289 | 0.039 | 177.389 | 166.868, 187.910 | 0.019 | 2.995 | 0.003 | -83.0 | 185.8 |
| L_0.75,T,PGLS_ | 5 | -11.376 | -11.470, -11.282 | <10^-6^ |  |  |  |  |  |  | 379.805 | 377.243, 382.367 | <10^-7^ | 3.263 | 0.060 | *+inf* | *+inf* |
| L_0.67,T,PGLS_ | 5 | -9.429 | -14.668, -4.190 | 0.039 |  |  |  |  |  |  | 324.961 | 153.022, 496.900 | 0.034 | 1.785 | 0.097 | *+inf* | *+inf* |

Table S19. Metabolic scaling in Carnivora. Data set on Carnivora is a subset from Sieg *et al.* (2009). It comprises those species for which temperature and phylogenetic information is available. Estimated beta values (ß.), their 95% confidence intervals (95%CI), their p-values, residual standard errors of models (Res.Se.), AICc and ΔAIC values of models are listed for all 16 models studied. ß_0_ = intercept, ß_1_ = slope, ß_2_ = coefficient of the quadratic term, ß_3_ = coefficient of the temperature term, λ = strength of the phylogenetic signal (only PGLS). *n.s*.: non-significant model, *div*: no convergence of fitting, *inf.*: infinity.

| model | N | *ß_0_* | 95%CI | p | *ß_1_* | 95%CI | p | *ß_2_* | 95%CI | p | *ß_3_* | 95%CI | p | λ | Res.S.E. | AICc | ΔAIC |
| --- | --- | --- | --- | --- | --- | --- | --- | --- | --- | --- | --- | --- | --- | --- | --- | --- | --- |
| L | 43 | 0.561 | 0.273, 0.849 | <10^-3^ | 0.725 | 0.647, 0.803 | <10^-16^ |  |  |  |  |  |  |  | 0.183 | -19.6 | 10.2 |
| C | 43 | 2.131 | 1.222, 3.040 | <10^-4^ | -0.179 | -0.687, 0.329 | 0.494 | 0.125 | 0.054, 0.196 | 0.001 |  |  |  |  | 0.162 | -28.8 | 1.0 |
| L_0.75_ | 43 | 0.470 | 0.415, 0.525 | <10^-16^ |  |  |  |  |  |  |  |  |  |  | 0.181 | -21.5 | 8.3 |
| L_0.67_ | 43 | 0.755 | 0.700, 0.810 | <10^-16^ |  |  |  |  |  |  |  |  |  |  | 0.184 | -20.1 | 9.7 |
| L_T_ | 43 | 2.763 | 0.958, 4.568 | 0.005 | 0.761 | 0.681, 0.841 | <10^-16^ |  |  |  | -88.051 | -159.362, -16.740 | 0.020 |  | 0.173 | -23.1 | 6.7 |
| C_T_ | 43 | 3.403 | 1.702, 5.104 | <10^-3^ | -0.030 | -0.553, 0.493 | 0.910 | 0.108 | 0.037, 0.179 | 0.005 | -59.452 | -127.229, 8.325 | 0.093 |  | 0.158 | -29.4 | *n.s.* |
| L_0.75,T_ | 43 | 2.711 | 0.971, 4.451 | 0.004 |  |  |  |  |  |  | -84.658 | -150.351, -18.965 | 0.016 |  | 0.171 | -25.4 | 4.4 |
| L_0.67,T_ | 43 | 2.314 | 0.474, 4.154 | 0.018 |  |  |  |  |  |  | -58.866 | -128.402, 10.671 | 0.105 |  | 0.181 | -20.6 | *n.s.* |
| L_PGLS_ | 43 | 0.330 | -0.021, 0.681 | 0.073 | 0.787 | 0.705, 0.869 | <10^-16^ |  |  |  |  |  |  | 0.847 | 0.227 | -28.1 | 1.7 |
| C_PGLS_ | 43 |  |  |  |  |  |  |  |  |  |  |  |  |  |  |  | *div.* |
| L_0.75,PGLS_ | 43 | 0.463 | 0.285, 0.641 | <10^-5^ |  |  |  |  |  |  |  |  |  | 0.806 | 0.218 | -29.8 | **0** |
| L_0.67,PGLS_ | 43 | 0.754 | 0.587, 0.921 | <10^-10^ |  |  |  |  |  |  |  |  |  | 0.700 | 0.218 | -23.4 | 6.4 |
| L_T,PGLS_ | 43 | 1.975 | 0.276, 3.674 | 0.028 | 0.801 | 0.721, 0.881 | <10^-16^ |  |  |  | -63.527 | -128.777, 1.723 | 0.064 | 0.771 | 0.207 | -29.1 | *n.s.* |
| C_T,PGLS_ | 43 |  |  |  |  |  |  |  |  |  |  |  |  |  |  |  | *div.* |
| L_0.75,T,PGLS_ | 43 | 1.919 | 0.228, 3.610 | 0.032 |  |  |  |  |  |  | -54.507 | -117.595, 8.581 | 0.098 | 0.724 | 0.201 | -30.1 | *n.s.* |
| L_0.67,T,PGLS_ | 43 | 1.771 | -0.069, 3.611 | 0.066 |  |  |  |  |  |  | -38.127 | -106.823, 30.569 | 0.283 | 0.654 | 0.211 | -22.2 | *n.s.* |

Table S20. Metabolic scaling in Artiodactyla. Data set on Artiodactyla is a subset from Sieg *et al.* (2009). It comprises those species for which temperature and phylogenetic information is available. Estimated beta values (ß.), their 95% confidence intervals (95%CI), their p-values, residual standard errors of models (Res.Se.), AICc and ΔAIC values of models are listed for all 16 models studied. ß_0_ = intercept, ß_1_ = slope, ß_2_ = coefficient of the quadratic term, ß_3_ = coefficient of the temperature term, λ = strength of the phylogenetic signal (only PGLS). *n.s*.: non-significant model, *div*: no convergence of fitting, *inf.*: infinity.

| model | N | *ß_0_* | 95%CI | p | *ß_1_* | 95%CI | p | *ß_2_* | 95%CI | p | *ß_3_* | 95%CI | p | λ | Res.S.E. | AICc | ΔAIC |
| --- | --- | --- | --- | --- | --- | --- | --- | --- | --- | --- | --- | --- | --- | --- | --- | --- | --- |
| L | 8 | 0.496 | 0.271, 0.721 | 0.005 | 0.762 | 0.711, 0.813 | <10^-6^ |  |  |  |  |  |  |  | 0.052 | -15.0 | 27.1 |
| C | 8 | 1.059 | -0.213, 2.331 | 0.164 | 0.497 | -0.093, 1.087 | 0.160 | 0.030 | -0.037, 0.097 | 0.418 |  |  |  |  | 0.089 | -6.9 | *n.s.* |
| L_0.75_ | 8 | 0.590 | 0.557, 0.582 | <10^-8^ |  |  |  |  |  |  |  |  |  |  | 0.049 | -20.4 | 21.7 |
| L_0.67_ | 8 | 0.897 | 0.838, 0.956 | <10^-7^ |  |  |  |  |  |  |  |  |  |  | 0.084 | -11.7 | 30.4 |
| L_T_ | 8 | 0.040 | -1.565, 1.645 | 0.963 | 0.768 | 0.709, 0.827 | <10^-7^ |  |  |  | 16.467 | -40.826, 73.760 | 0.598 |  | 0.055 | -6.2 | *n.s.* |
| C_T_ | 8 | 0.632 | -1.630, 2.894 | 0.613 | 0.517 | -0.132, 1.166 | 0.193 | 0.029 | -0.045, 0.103 | 0.488 | 14.350 | -45.736, 74.436 | 0.664 |  | 0.057 | -11.4 | *n.s.* |
| L_0.75,T_ | 8 | 0.286 | -1.033, 1.605 | 0.685 |  |  |  |  |  |  | 10.062 | -40.486, 60.610 | 0.710 |  | 0.052 | -15.0 | *n.s.* |
| L_0.67,T_ | 8 | 1.374 | -0.892, 3.640 | 0.280 |  |  |  |  |  |  | -18.247 | -105.069, 68.575 | 0.695 |  | 0.089 | -6.3 | *n.s.* |
| L_PGLS_ | 8 | 0.239 | -0.027, 0.451 | 0.068 | 0.828 | 0.828, 0.828 | <10^-16^ |  |  |  |  |  |  | 1.322 | 0.172 | -13.0 | 29.1 |
| C_PGLS_ | 8 |  |  |  |  |  |  |  |  |  |  |  |  |  |  |  | *div.* |
| L_0.75,PGLS_ | 8 | 0.545 | 0.545, 0.545 | <10^-16^ |  |  |  |  |  |  |  |  |  | -0.303 | 0.045 | -42.1 | **0** |
| L_0.67,PGLS_ | 8 | 0.899 | 0.899, 0.899 | <10^-16^ |  |  |  |  |  |  |  |  |  | -0.528 | 0.071 | -21.9 | 20.2 |
| L_T,PGLS_ | 8 |  |  |  |  |  |  |  |  |  |  |  |  |  |  |  | *div.* |
| C_T,PGLS_ | 8 |  |  |  |  |  |  |  |  |  |  |  |  |  |  |  | *div.* |
| L_0.75,T,PGLS_ | 8 |  |  |  |  |  |  |  |  |  |  |  |  |  |  |  | *div.* |
| L_0.67,T,PGLS_ | 8 |  |  |  |  |  |  |  |  |  |  |  |  |  |  |  | *div.* |

Table S21. Median, minimum and maximum of log_10_ body masses (BM), of body temperatures (T) and log_10_ basal metabolic rates (BMR) of taxa studied.

| Taxon | N | Median BM | Min BM | Max BM | Median T | Min T | Max T | Median BMR | Min BMR | Max BMR |
| --- | --- | --- | --- | --- | --- | --- | --- | --- | --- | --- |
| All mammals | 519 | 1.994 | 0.380 | 5.557 | 36.5 | 30.5 | 40.7 | 2.044 | 0.785 | 4.766 |
| Marsupialia | 63 | 2.824 | 0.806 | 4.477 | 35.3 | 32.5 | 37.4 | 2.504 | 1.029 | 3.764 |
| Eutheria | 456 | 1.885 | 0.380 | 5.557 | 36.8 | 30.5 | 40.7 | 1.988 | 0.785 | 4.766 |
| Dasyuromorphia | 21 | 1.652 | 0.806 | 3.788 | 35.2 | 32.5 | 37.4 | 1.805 | 1.029 | 3.225 |
| Didelphimorphia | 11 | 2.536 | 1.161 | 3.396 | 35.0 | 32.6 | 35.8 | 2.343 | 1.417 | 2.904 |
| Peramelemorphia | 8 | 2.923 | 2.530 | 3.079 | 35.1 | 33.8 | 36.1 | 2.575 | 2.074 | 2.679 |
| Diprotodontia | 23 | 3.300 | 1.000 | 4.477 | 35.9 | 33.7 | 37.4 | 2.841 | 1.179 | 3.763 |
| Soricomorpha | 23 | 1.009 | 0.380 | 1.690 | 37.0 | 34.3 | 38.7 | 1.493 | 1.097 | 2.043 |
| Chiroptera | 73 | 1.340 | 0.690 | 3.010 | 35.9 | 30.5 | 38.8 | 1.496 | 0.785 | 2.905 |
| Macroscelidea | 8 | 1.682 | 1.589 | 2.316 | 37.3 | 36.2 | 37.6 | 1.725 | 1.640 | 2.231 |
| Afrosoricida | 9 | 1.646 | 0.839 | 2.857 | 32.8 | 30.8 | 34.0 | 1.637 | 0.886 | 2.374 |
| Rodentia | 236 | 1.838 | 0.863 | 4.422 | 37.0 | 32.1 | 40.7 | 1.947 | 1.130 | 3.820 |
| Erinaceomorpha | 7 | 2.656 | 1.890 | 2.972 | 35.2 | 33.8 | 37.3 | 2.179 | 1.907 | 2.703 |
| Primates | 18 | 2.950 | 1.752 | 4.201 | 37.0 | 33.8 | 39.3 | 2.530 | 1.885 | 3.705 |
| Lagomorpha | 11 | 3.332 | 2.039 | 3.520 | 38.9 | 37.7 | 40.1 | 3.088 | 2.229 | 3.354 |
| Pilosa | 6 | 3.551 | 2.355 | 4.486 | 33.0 | 32.0 | 34.4 | 2.916 | 2.052 | 3.415 |
| Cingulata | 9 | 3.552 | 3.045 | 4.655 | 34.4 | 33.0 | 35.5 | 2.907 | 2.322 | 3.481 |
| Pholidata | 5 | 3.561 | 3.155 | 4.202 | 33.0 | 32.3 | 33.4 | 2.825 | 2.574 | 3.094 |
| Carnivora | 43 | 3.560 | 1.906 | 5.140 | 38.0 | 34.6 | 39.8 | 3.052 | 2.270 | 4.380 |
| Artiodactyla | 8 | 4.408 | 3.208 | 5.557 | 38.6 | 36.1 | 39.4 | 3.814 | 2.950 | 4.766 |
